# A Combined Experimental and Modeling Workflow to Tune Surface Properties of Organic Materials via Cocrystallization

**DOI:** 10.1021/acs.chemmater.5c00634

**Published:** 2025-07-17

**Authors:** Emmanuele Parisi, Giulia Del Duca, Emilia Prandini, Silvia Fraterrigo Garofalo, Chiara Rosso, Michele Remo Chierotti, Elena Simone

**Affiliations:** † Department of Applied Science and Technology (DISAT), 19032 Politecnico di Torino, Corso Duca Degli Abruzzi 24, Torino I-10129, Italy; ‡ Department of Chemistry and NIS Centre, 9314 University of Torino, Via P. Giuria 7, Torino I-10125, Italy

## Abstract

Cocrystallization is a specific crystal engineering strategy widely used to enhance the dissolution rate or bioavailability of active pharmaceutical ingredients. In this work, we demonstrate how cocrystallization can also be used to tune surface properties of crystalline particles, such as facet-specific surface chemistry, polarity, and wettability. As a model system, we have isolated a cocrystal of quercetin (Que) with imidazole (Im). Que is widely recognized for its potential antioxidative and antibacterial properties and other potentially beneficial therapeutic effects. Surface chemistry is a property that can affect ease of manufacturability (e.g., flowability) and storage stability (e.g., tendency to agglomerate) for particulate materials; here, we used cocrystallization to modify this property for Que particles. The screening of suitable coformers was first performed in silico using a method based on molecular complementarity and hydrogen bond (H-bond) propensity scores. Experiments were conducted using the identified coformers via slurrying in different solvents. The cocrystal was identified and characterized by powder X-ray diffraction (PXRD), differential scanning calorimetry (DSC), thermogravimetric analysis (TGA), Raman spectroscopy, and solid-state nuclear magnetic resonance (SSNMR). The Que-Im crystal structure was solved by single-crystal X-ray diffraction (SXRD) and characterized computationally, using the attachment energy model, and experimentally by contact angle measurements. Structural and vibrational analyses showed a major modification in intermolecular interactions of Que-Im compared to pure Que polymorphs. The contribution of the H-bond and π–π stacking interactions to the crystal energy is similar, but the crystal morphology exposes a predominant facet growing via van der Waals interactions. As a result, Que-Im is more hydrophobic than the dihydrate (QDH) and dimethyl sulfoxide (QDMSO) solvate forms. The shift in the average water droplet contact angle from 38.8 ± 1.0° (QDMSO), 48.0 ± 3.2° (QDH) to 78.5 ± 3.9° (Que-Im) is strong evidence of a marked decrease in hydrophilicity of the target compound.

## Graphical abstract

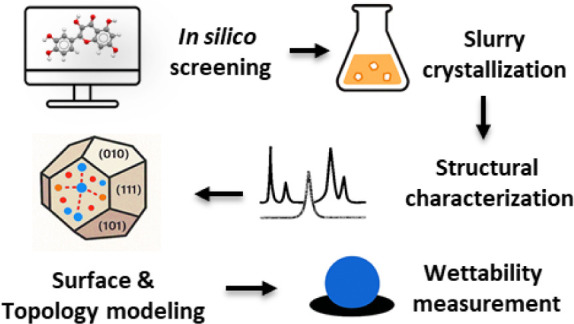

## Introduction

It is well known how and to what extent properties such as chemical and molecular composition, crystal size and shape distribution, and internal crystal structure (e.g., polymorphism) determine key performance features of particulate materials in terms of stability, texture, solubility, and dissolution rates.^1–3^ However, the effect of surface and interfacial properties on the quality and manufacturability of particulate products is less understood. The complex nature of surfaces in organic crystalline particles (including the intrinsic anisotropy of molecular crystals) and the lack of suitable surface analysis techniques for the characterization of facet-specific properties of micrometer-sized particles are some of the reasons for this gap in knowledge. Nevertheless, surface properties are known to impact several steps of manufacturing, such as blending, milling, granulation, and tableting, as well as product performance, like stability, powder caking, or dissolution profiles.^4,5^ Hence, developing experimental and computational strategies to measure, calculate, and tailor surface properties of crystalline particles is essential to improve product and process development as well as manufacturing efficiency.

The crystal engineering approach, based on understanding supramolecular interactions in crystal packing, has been used to design various solid forms—cocrystals, salts, solvates, hydrates, and polymorphs—of target molecules to fine-tune bulk properties and develop organic and inorganic–organic frameworks with tailored functionalities and desired physicochemical properties, such as solubility, dissolution rate, and bioavailability.^6^ These crystal forms find use as advanced materials for pharmaceutical, nutraceutical, and agrochemical applications.^7–21^ Additionally, the identification and/or design of supramolecular synthons that are strong enough to be exchanged from one network structure to another provides structural predictability for the isolation of novel solid forms.^22^ In this way, supramolecular architectures can be finely designed by the selection of specific supramolecular interactions.

Cocrystallization represents the fastest-growing and most widespread approach for designing organic crystalline structures.^23–26^ Alongside advances in experimental screening techniques,^27,28^ virtual screening methods have been developed to ease the synthesis of cocrystals. These methods are based on predicting the propensity for interaction^29,30^ or electrostatic potentials^31^ between molecules, or on molecular complementarity.^32^ Machine learning approaches based on molecular descriptor similarities have also been developed to predict suitable coformers.^33–35^ These computational tools aim to reduce the time and chemical resources required for experimental cocrystal screening, speeding up the discovery of novel solid forms. However, significant challenges remain in predicting *a priori* which crystal structure features drive specific particle properties, particularly surface and interfacial ones. Indeed, a deep understanding of the relationship between the crystal structure and facet-specific surface properties is still missing and represents the bottleneck in the application of crystal engineering in this area of research. Some work has been done to estimate solvent–surface interactions and surface–surface interaction energies, which affect product quality and manufacturability (e.g., storage stability, flowability, compressibility),^36–45^ but we are still far from a precise design of surface properties using the crystal engineering approach.

In this study, the possibility of tuning surface properties of quercetin (Que) (Figure 1) using cocrystallization was investigated. To achieve this, cocrystal screening tools, such as molecular complementarity analysis (MC)^32^ and the hydrogen-bond propensity (HBP) method,^46^ were combined with experimental (e.g., contact angle measurements) and computational techniques (lattice energy calculations, synthon analysis, and surface termination estimation) for the characterization of surface properties of crystals, such as facet-specific rugosity and chemistry. The proposed multitechnique methodology could be extended to other organic molecules with the aim of correlating the crystal structure to facet-specific surface features (i.e., exposure of different functional groups on the facet termination, roughness) and then to particle properties (i.e., hydrophilicity/hydrophobicity, wettability).

**Figure 1:**
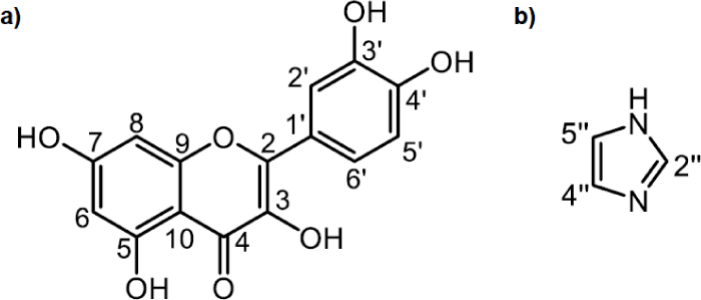
Molecular diagram of a) quercetin (Que) and b) imidazole (Im) with atom numbering.

Que, 2-(3,4-dihydroxyphenyl)-3,5,7-trihydroxy-4*H*-chromen-4-one, is a nutraceutical flavonoid found in various fruits and vegetables, including tomatoes, berries, citrus fruits, and onions.^47,48^ It is renowned for its potential antibacterial and antioxidative properties and a range of other advantageous therapeutic benefits, including anti-inflammatory, antiviral, anticancer, metal chelation, and cardioprotective effects.^49–53^ Que shows a fascinating solid-state landscape, including several anhydrous (two polymorphic forms), hydrates (mono/dihydrate) and solvate forms (dimethylformamide and dimethyl sulfoxide), whose relative stability, crystallization behavior, and the physicochemical properties are strongly related to the molecular conformation in the crystal lattice and the crystal packing modes.^44,45,47,54–56^ Through synthon analysis, we previously showed how surface properties are related to the crystal structure of Que.^43^ Here, we focus on developing a combined experimental and computational workflow for the use of cocrystallization to tailor Que’s surface properties.

## Experimental Section

### Materials

Que dihydrate (QDH) with a purity of 97% was obtained from Alfa Aesar (Port of Heysham Industrial Park, Lancashire, England), while imidazole (Im) with a purity of 99% was obtained from Thermo Fisher Scientific Chemicals. Ferulic acid (FA) and 4-aminobenzoic acid (PABA) with a purity of 99% were obtained from Sigma-Aldrich. Methanol (MeOH), ethanol (EtOH), acetonitrile (MeCN), and 2-propanol 99.5% (IPA) were obtained from VWR. Dimethyl sulfoxide 99.9% (DMSO) was obtained from Sigma-Aldrich. Water purified by treatment with a Milli-Q apparatus was used for all of the experiments.

### Computational Procedures

MC is a method for assessing the likelihood of two molecules to form a cocrystal. A survey conducted on structures of cocrystals deposited in the Cambridge Structural Database (CSD) indicates that the molecular polarity, shape, and size of molecules that cocrystallize together tend to be similar.^32^ A set of 93 potential coformers (listed in the Table S1) was selected, converted to .mol2 files, and imported into Mercury 2023.3.1 software^57^ (CSD, Cambridge, UK) for the MC analysis (molecular complementarity screening tool).^32^ For cocrystallization to be likely, five key molecular descriptors have been identified, for which the difference between the values for the two cocrystal components should be small. Three shape descriptors based on the molecular bounding box (the length of the short axis, the short/long axis ratio, and the medium/long axis ratio) and two polar descriptors (fraction of N and O atoms and dipole moment magnitude) were used to evaluate the similarity of the molecules. Each descriptor outputs a “PASS” or “FAIL” signal, with “PASS” indicating a 100% hit rate, signifying the potential formation of multicomponent crystals, whereas “FAIL” corresponds to a 0% hit rate, indicating the inability to form multicomponent crystals. The 56 coformers (Table S2) that passed the MC screening were ranked based on their MCHB propensity scores, which were calculated using the Mercury 2023.3.1 software.^58^ The “Multi_component_hydrogen_bond_propensity_report” Python script and standard settings are available on GitHub.^59^ The propensity of the highest heteromeric interaction between Que and a coformer (C) (*P*_Que–C_) was compared with the highest homomeric interaction, either Que–Que (*P*_Que–Que_) or coformer–coformer (*P*_C–C_). The difference Δ_HBP_ was used to estimate the likelihood of cocrystallization, with higher values indicating a greater likelihood of cocrystallization between the two molecules.^46^ This difference is calculated as

1 $$\Delta_{HBP}=P_{Que-C}-[\max\left(P_{Que-Que},P_{C-C}\right)$$

The structure minimization and the unit cell parameter optimization of the Que-Im cocrystal were performed using the Forcite module in Materials Studio 2021 (v21.1.1.3268). The torsion angle between the phenyl and pyrone rings was kept rigid. The SMART algorithm was selected for the structural minimization, and the Dreiding force field was used. General force constants and geometry parameters for this force field are based on simple hybridization rules rather than on specific combinations of atoms. The van der Waals interactions are described by the Lennard-Jones potential. Electrostatic interactions are described by atomic monopoles and a distance-dependent Coulombic term. Hydrogen bonding is described by an explicit Lennard-Jones 12–10 potential. The optimization parameters are reported in the Supporting Information (Geometry optimization section). The intrinsic synthon analysis and calculation of the intermolecular interactions in the studied crystal structures were carried out with the Visual Habit function in the CSD Particle tool in Mercury, considering a fixed molecule and all of the other molecules within a distance of 30 Å. In the calculation, the Dreiding II force field was used. The contributions per functional group and per atom type to the total lattice energy of each structure were calculated by using the DEBUG-2 function and were summed over the asymmetric unit. The ranking of the intermolecular interactions by strength was output by using the DEBUG-1 function. The surface topology was calculated on the basis of the morphology predicted by the attachment energy model. Nevertheless, simulated results were interpreted based on the experimental morphology obtained via X-ray analytical indexing. The attachment energy model (implemented in the CSD-Particle, Surface Analysis tool in Mercury) was applied to calculate the specific synthons contributing to the growth of the main facets, the contributions of polar and nonpolar interactions to the facet-specific attachment energies, and to simulate facet-specific topology (rugosity) and chemical nature (bond density per unit area of hydrogen-bond donors (HBDs), hydrogen-bond acceptors (HBAs), aromatic groups). Surfaces and morphologies were visualized using CCDC’s Mercury 2023.3 software. Surfaces were defined using the procedure described by Prandini et al.^41^

### Preparation of the Quercetin-Imidazole Cocrystal (Que-Im) and Quercetin-DMSO (QDMSO) Solvate

6.00 g portion of QDH (17.7 mmol) and 0.604 g of Im (8.87 mmol) were introduced with 100 mL of MeCN into a 250 mL jacketed vessel and slurried for 4 days at 25 °C. The temperature was controlled with a Huber Ministat 230 connected to a PT probe inserted into the vessel. The solid was recovered by vacuum filtration and air-dried overnight on a paper filter, yielding 5.07 g of pale-yellow powder in a stoichiometric ratio of 2:1. The recovery yield was around 85% of the total mass introduced into the vessel. The purity of the powder was checked with DSC and NMR. Melting point: 313 °C. ^1^H NMR (600.17 MHz, DMSO-*d*_6_): d 6.18 (s, 1H), 6.40 (s, 1H), 6.88 (d, 2H), 7.02 (d, 2H), 7.53 (d, 2H), 7.65 (s, 1H), 7.67 (s, 1H).

Single crystals of Que-Im were grown by using the evaporation setup on the CrystalBreeder platform (Technobis Crystallization Systems, The Netherlands). 0.1 mL portion of a 0.1 M 2-propanol solution of Que-Im was heated to 35 °C, and the solvent was slowly evaporated with a vacuum pump set at 150 mbar for 24 h.

QDMSO synthesis and crystallization were performed by following the experimental procedure reported in the literature.^45^

### Slurry Crystallization Experiments of Que with PABA and FA

500 mg of QDH (1.4 mmol) were slurried for 7 days at 25 °C, 30 °C, 35 °C, and 40 °C in the Crystal16 platform (Technobis Crystallization Systems, The Netherlands) with 1:1, 1:2, and 2:1 stoichiometric ratios of FA and 1 mL of EtOH/MeOH/IPA/MeCN. Bottom stirring at a fixed speed of 780 rpm was used to keep the particles well dispersed. The same set of experiments was performed with PABA.

### Thermogravimetric Analysis and Differential Scanning Calorimetry (TGA and DSC)

The thermal properties were studied by using a Mettler Toledo 8000 DSC-1 calorimeter. The differential scanning calorimeter was calibrated by using indium. The samples were heated from 25 to 350 °C at a heating rate of 10 °C/min. Nitrogen was used as the purge gas at a rate of 50 mL/min. The heat flow was measured in mW, and the melting point of the sample was determined through peak height. A Mettler Toledo TGA instrument (1600, Columbus, OH, USA) was employed for thermogravimetric analysis. The sample was heated with a constant heating ramp of 10 °C/min from 25 to 500 °C. Argon (Ar) was supplied at a constant flow rate of 50 mL/min.

### X-Ray Diffraction (PXRD, VT-PXRD, SXRD)

X-ray powder patterns of the powdered samples were acquired on a PANalytical X’Pert Pro in the Bragg–Brentano geometry, using Cu Kα X-radiation (λ = 1.54506 Å) at 40.0 kV and 40.0 mA. The measurements were collected in θ/2θ mode over the 2θ range 3°–40° with a step size of 0.01313° (2θ) and a time per step of 30 s. Samples were prepared on a Si zero background and measured without spinning. Furthermore, to study the stability of the cocrystal, variable temperature powder X-ray diffraction (VT-PXRD) measurements were performed using a PANalytical Empyrean X-ray diffractometer, equipped with an HTK1200N (Anton Paar) hot chamber, a sealed tube copper X-ray source (Ni-filtered Cu Kα radiation, λ = 1.54178 Å), and fitted with a PixCel3D Medipix detector. The powder sample was loaded into an aluminum oxide sample holder, and the temperature was increased in steps from 25 to 330 °C at a rate of 10 °C min^–1^. The measurements were performed in reflection mode geometry with a step size of 0.01313° (2θ) and a time per step of 150 s. Single-crystal data were collected on a Gemini R Ultra diffractometer (Agilent Technologies UK Ltd., Oxford, U.K.) using Cu Kα radiation (λ = 1.5406 Å) with the ω-scan method. CrysAlisPro software (v.171.42.49, Rigaku Oxford Diffraction) was used for retrieving cell parameters, performing data reduction, and analytical absorption correction (with the multi-scan technique). The structure was solved with direct methods using ShelXS-2016 and refined with full-matrix least-squares on F^2^ using SHELXL-2016,^60^ both operating under the Olex2.1–5 program.^61^ All non-hydrogen atoms were anisotropically refined. Hydrogen atoms bonded to O and N were placed in calculated positions and refined by the riding model. For all the H atoms, U_iso_= 1.2 x U_eq_ of the carrier atom was assumed. Images of the structures were obtained by using Mercury software. Crystal data and refinement details, selected bond lengths and angles, amplitudes, and asymmetric units of the compounds are reported in the Supporting Information. The crystallographic data are deposited in the Cambridge Crystallographic Data Centre as CCDC number 2405458. This information can be obtained free of charge from the Cambridge Crystallographic Data Centre via www.ccdc.cam.ac.uk/data_request/cifcodeCCDC.

### Raman Spectroscopy

Raman spectra of samples were acquired by using a 785 nm laser source with a LabRAM HR Evolution spectrometer (HORIBA Scientific, France) equipped with a 50× LWD objective. Backscattered radiation was collected with a Synapse Plus BIDD detector (1024 pixels × 256 pixels), utilizing a 300 l/nm grating. The laser power was set to 100%. Spectra were acquired with a 5 s acquisition time for 5 accumulations.

### Solid-State NMR Spectroscopy

^13^C and ^15^N CPMAS (cross-polarization magic angle spinning) SSNMR spectra were acquired with a Bruker Avance II 400 Ultra Shield instrument, operating at 400.23, 100.63, and 40.56 MHz for ^1^H, ^13^C, and ^15^N nuclei, respectively. The powder samples without further preparations or treatments were packed into cylindrical zirconia rotors with a 4 mm o.d. and 80 μL volume. ^13^C and ^15^N CPMAS spectra were acquired at room temperature at spinning speeds of 12 and 9 kHz, respectively, using a ramp cross-polarization (CP) pulse sequence with a ^1^H 90° pulse of 3.80 μs, a contact time of 3 ms for ^13^C and 4 ms for ^15^N, an acquisition time of 29.99 ms for ^13^C and 34.94 ms for ^15^N, optimized recycle delays (^1^H T_1_ * 1.27) of 6.33 s, and a number of scans equal to 4210 for ^13^C and 40 200 for ^15^N. The ^13^C NQS (non-quaternary suppression) spectrum was acquired under the same operating conditions as the ^13^C CPMAS spectra, using a delay of 45 μs before the acquisition, a ^13^C 180° refocusing pulse of 8 μs, and a number of scans of 3000. A two-pulse phase modulation (TPPM) scheme was used for heteronuclear decoupling, with a radio frequency field of 65.8 kHz. The ^13^C and ^15^N chemical shift scales were calibrated through the methyl signal of the external standard adamantane (δ­(^13^C) 38.48 ppm with respect to tetramethylsilane, TMS) and the NH_3_^+^ signal of the external standard α-glycine (δ­(^15^N) 33.4 ppm with respect to liquid NH_3_). The solution ^1^H NMR experiment was performed on a Jeol ECZR 600 instrument operating at 600.17 MHz for ^1^H. The spectrum was acquired in DMSO-*d*_6_ at 25 °C, by using a relaxation delay of 150 s and a number of scans of 256.

### Solubility Measurements

The Crystal16 platform (Technobis Crystallization Systems, The Netherlands) was used to determine the solubility of the QDH, Im, and Que-Im samples. Isopropanol was used as a reference solvent. In Crystal16, the dissolution temperatures of slurries contained in 1 mL stirred vials can be measured in parallel and automatically, based on the value of the turbidity. Bottom stirring at a fixed speed of 780 rpm was used to keep the particles well dispersed. The samples were analyzed by changing the temperature from 20 to 70 °C, with a heating rate of 0.3 °C/min and a cooling rate of −0.3 °C/min.

### Scanning Electron Microscopy (SEM)

Before carrying out the contact angle measurements and verifying the consistency with the results from SCXRD indexing, the morphologies of Que-Im, together with particles of QDH and QDMSO, were observed with SEM. This measurement was carried out to examine the particles’ morphology and confirm consistency with the results from SCXRD indexing. Particles were placed on stubs with carbon tape and coated with platinum at 30 mA for 30 s. The images were collected with a Quanta 3D FEG 200i operating at a 30 kV voltage and at different magnifications (800× and 2 k×).

### Contact Angle Measurements

The wettability of the samples was evaluated by measuring the contact angle between a disk of compressed powder and a droplet of Milli-Q water. The measurements were carried out at room temperature using a DSA25 Drop Shape Analyzer (Krüss Scientific) fitted with a microsyringe and a CF30 high-speed camera with a CMOS sensor. Compressed disks of powders of approximately 100 mg were made by placing gently ground powder samples between the plates of a hydraulic bench press with a diameter of 1.2 cm. Two trace paper disks were placed between the plates to ensure the formation of a homogeneous and smooth disk surface. The powder was then pressed under a weight of 200 bar for 30 s. Static contact angles were measured by using the sessile drop method. Water droplets (2 μL) were produced by using a straight needle to form a sessile drop onto the compressed particle disk surfaces. A high-speed camera was used to record the droplet behavior. The droplet contour was fitted using the Young–Laplace method with Krüss Advance 1.12.0.35401 software, and the contact angles between the disk and the water (θw) droplet were determined. All measurements were repeated 6 times. It is worth noting that the porosity and roughness of the disks may affect the measurements. To minimize the effect of these parameters, we: (1) used the same relatively high pressure to prepare all disks; (2) measured different droplets in different regions of the same disk; and (3) used SEM to verify the morphology of the measured particles, as needles and plates can form more compact and smooth powder disks.

## Results and Discussion

### Virtual Cocrystal Screening

The MC analysis was the first tool used for cocrystal screening for Que. This method identifies suitable coformers based on molecular geometrical descriptors and polarity, specifically, the dipole moment magnitude and the fraction of N and O atoms in the considered molecules. As a geometrical descriptor, we used the two ratios among the three principal axis lengths of a rectangular box enclosing the van der Waals volume of the tested molecule.^32^ Based on these parameters, a ranking of the propensity of each coformer to form a cocrystal with Que was generated, considering those promising coformers with molecular shape and polarity parameters similar to Que. The PASS or FAIL thresholds are reported in the Supporting Information. The first screening of 93 selected coformer candidates, listed in Table S1, has returned 56 possible ones (Table S2, PASS), which were used for the second step of screening based on multicomponent hydrogen bond propensity (MCHBP). The first 10 ranked coformers obtained by the MCHBP analysis are reported in Table 1. This method analyzes the specific intermolecular interactions between Que and coformers and assumes that the strongest H-bond among all possible donor–acceptor pairs guides the formation of a crystal structure.^30,46,62^ The MCHB score calculated with this method ranges from +0.38 to −0.24 (Table 1). Overall, 18 Que:coformer combinations resulted in a positive value, as shown in Table 1, 6 with a 0 value, and the other combinations resulted in a negative value (Table S2). It is interesting to note that the highest MCHB score comes from the only two coformers that feature exclusively HBA groups, indicating a strong propensity of Que to act as an HBD rather than an HBA. Additionally, the first four coformers reported in Table 1 have nitrogen atoms with sp^2^ hybridization, which can be considered a favorable condition since many known Que cocrystals show this feature (e.g., CSD Refcodes LILLEB, MUPPOD, JATPIH, NAFYUR, QOLLUA, WISZAD, WISZEH, WISZIL).^11,63–66^ Among the coformers with positive MCHBP scores (Table 1), theophylline was previously reported to form a cocrystal with Que.^66^ From the fifth coformer onward, except for EDTA, the simultaneous absence of HBAs and the exclusive presence of HBDs complicates a supramolecular assembly with Que. This consideration can be supported by a qualitative study (a formal step in our in silico procedure, Chart [Fig chart1]) of the supramolecular interactions of Que crystal structures reported in the CSD (v. 5.46), in which it is evident that Que tends to form adducts where it acts as a strong HBD.^67^ Indeed, despite the negative scores obtained for isonicotinamide and nicotinamide (MC SCORE: −0.13 and −0.14, respectively), two cocrystals with Que are deposited in the CCDC, and in both cases, the coformers act as only HBAs.^65,68^ Considering the current limitations of these in silico tools, we decided to test experimentally three different coformers: Im, FA, and PABA. These molecules all show three different functionalities, which increases the possibility of forming cocrystals, and they are solid at room temperature. Im has the potential to act as both an HBD and an HBA, whereas FA and PABA are HBDs with different functional groups chosen as samples to confirm the structural characteristics mentioned above. In addition, the rational selection of the coformers was performed by considering possible interest from medicinal, nutraceutical, and pharmaceutical industries due to their high biological activity.^69–71^

**Table 1:** Results of MCHBP Analysis: Only Positive Multicomponent Scores

Rank	Component B	Multicomponent score
1	pyrazine	0.38
2	3-methylpyridine	0.36
3	theophylline	0.08
4	imidazole	0.07
5	2-amino-5-methylbenzoic_acid	0.06
6	ferulic acid	0.06
7	apigenin	0.05
8	ketoglutaric_acid	0.05
9	4-aminobenzoic_acid	0.03
10	EDTA	0.03
11	ethylparaben	0.02
12	hesperetin	0.02
13	propylparaben	0.02
14	d-pantothenol	0.01
15	methylparaben	0.01
16	glutaric_acid	0.01
17	glycolic_acid	0.01
18	l-mandelic_acid	0.01

**1 chart1:**
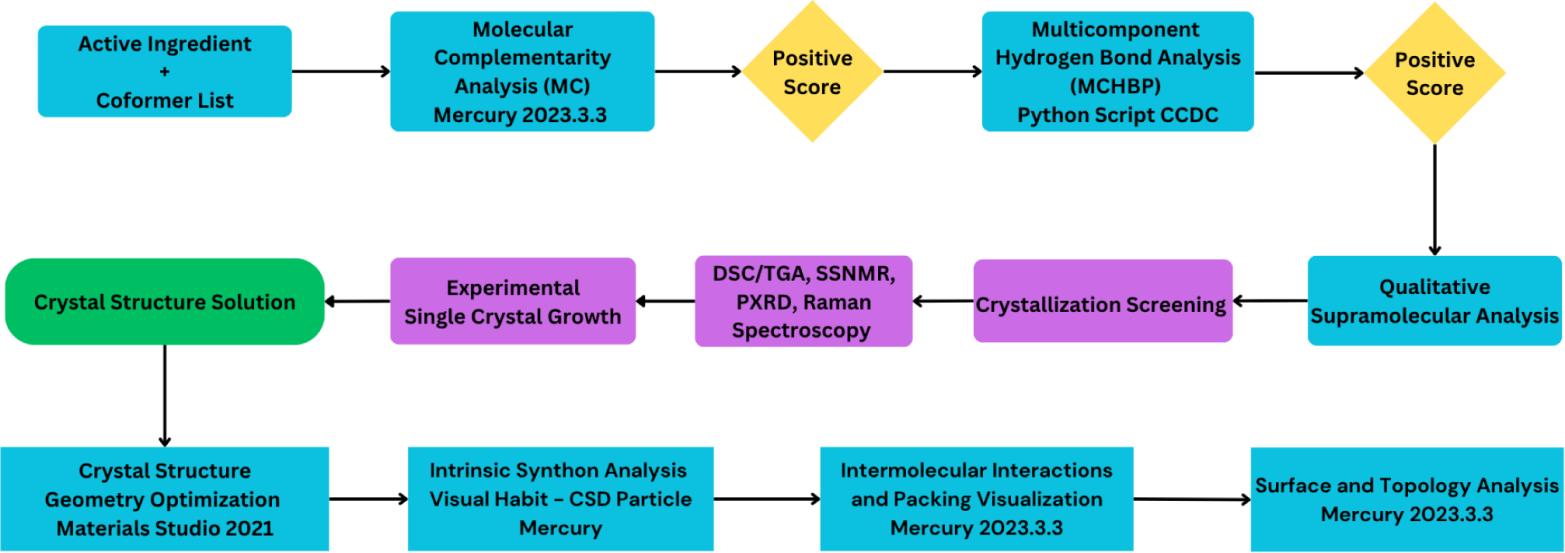
Computational and Experimental Workflow^a^

a Blue boxes for computational analysis, violet boxes for experimental crystallization and characterization, and green boxes for crystal structure solution.

### Cocrystallization Experiments and Characterization

Slurry and solution experiments with 1:1, 1:2, and 2:1 stoichiometric ratios of the Que and the selected coformers in different solvents were used for experimental cocrystal screening, based on the in silico screening results. The outcomes of the experiments were investigated by Raman spectroscopy and PXRD, and any potential cocrystals were further examined using DSC, TGA, and SSNMR analysis.

None of the cooling crystallization experiments led to the formation of cocrystals, probably due to the high solubility difference between Que and the coformers in the tested solvents. During cooling crystallization experiments, the least soluble component, Que, nucleated first, followed by the more soluble coformer. The PXRD and Raman data showed the presence of both pure components without any additional peaks. On the other hand, slurry cocrystallization experiments successfully led to the isolation of a cocrystal of Que with Im (Que-Im) with a stoichiometric ratio of 2:1. These results seem to confirm the hypothesis that Que tends to cocrystallize with molecules possessing acceptor nitrogen atoms to form strong H-bonds. Table 2 summarizes the outcomes of the slurry experiments.

**Table 2:** Results of the Slurry Experiments^a^

	Que:Im			Que:FA			Que:PABA		
Stoichiometric ratio	1:1	2:1	1:2	1:1	2:1	1:2	1:1	2:1	1:2
**EtOH**	NP	NP	NP	M	M	M	M	M	M
**IPA**	NP	P	P	M	M	M	M	M	M
**MeCN**	NP	P	P	M	M	M	M	M	M
**MeOH**	M	M	M	M	M	M	M	M	M
**Water**	M	M	M	M	M	M	M	M	M

a NP stands for not pure cocrystal phase, P stands for the pure cocrystal phase, and M stands for a mixture of pure starting materials.

The slurry experiments conducted in MeCN and IPA with 2:1 and 1:2 stoichiometric ratios successfully led to the isolation of the same cocrystal phase of Que-Im. Differently, the slurries performed in both IPA and MeCN with a 1:1 stoichiometric ratio led to the isolation of not pure phases. These experimental findings could be related to the higher solubility of Im in comparison to Que in the two solvents. For the slurry with a 1:2 stoichiometric ratio, the amount of Im that did not react remained in solution, providing in this way a pure cocrystal phase. On the other hand, the slurry with a 1:1 stoichiometric ratio did not contain enough Im to fully cocrystallize with Que, resulting in a mixture of Que-Im and pure QDH. For the other solvents used, the slurries led to the isolation of not pure phases or mixtures of the pure components, even for longer times of slurrying. Cocrystals were not identified for either FA or PABA. These results were confirmed by the Raman spectra and PXRD patterns, which have always shown mixtures of the starting materials (Figure S1). The experiments were also performed at higher temperatures (up to 40 °C) in order to reduce the activation energy of the cocrystallization processes, still with unsuccessful nucleation and growth of the cocrystals. In Figure 2, the diffraction pattern and the Raman spectrum of the Que-Im cocrystal form are reported and compared with those of the starting materials, showing clear differences in the intensity and position of the peaks.

**Figure 2:**
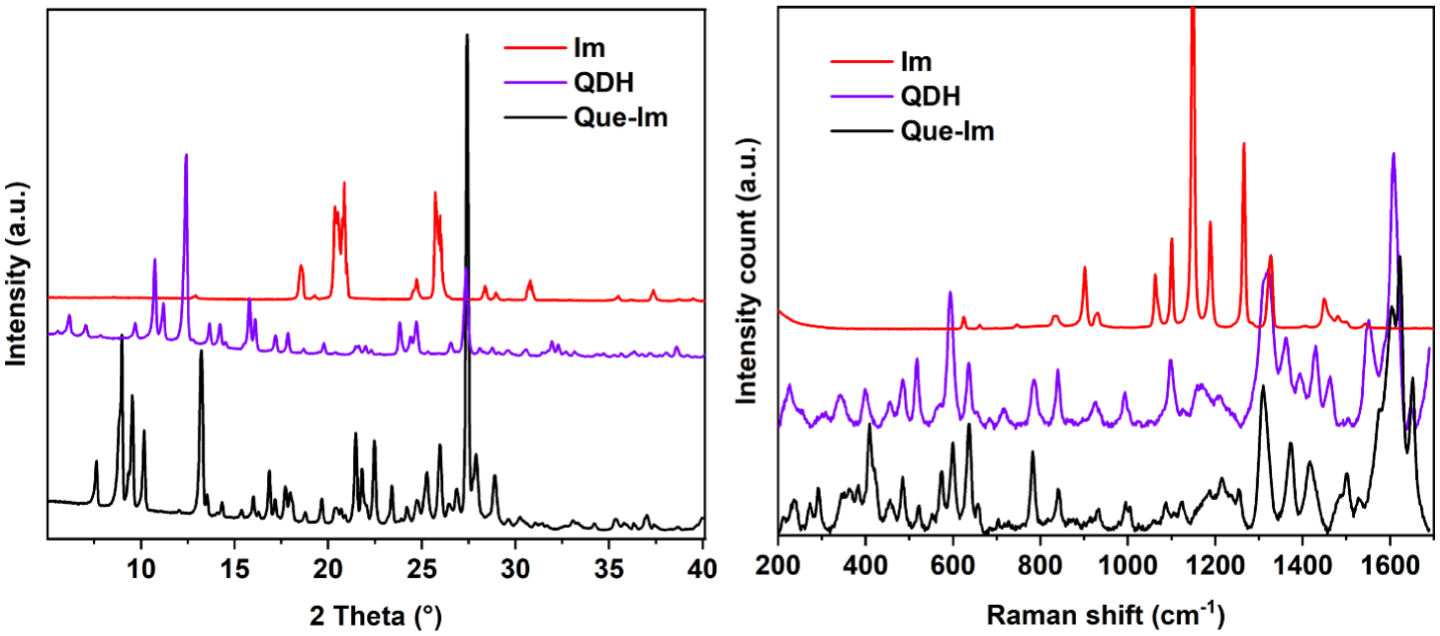
(Left) Comparison of the X-ray powder patterns of Que-Im and the starting materials QDH and Im. (Right) Comparison of the Raman spectra of Que-Im and the starting materials QDH and Im. Cocrystal (black), QDH (purple), and Im (red).

Comparing the Raman spectrum of Que-Im with those of the pure reagents, it is possible to note that the fingerprint area below 1000 cm^–1^, which is associated with the ring stretching of Que, is almost identical to that of pure Que.^72^ In contrast, the region between 1300 and 1700 cm^–1^ presents shifted peaks associated with the stretching of the C=O carboxylic group and bending of OH groups of Que that are involved in strong H-bond interactions with Im.^73^ The thermal analyses, DSC and TGA, were carried out for the Que-Im cocrystal and compared with the starting materials (Figure 3).

**Figure 3:**
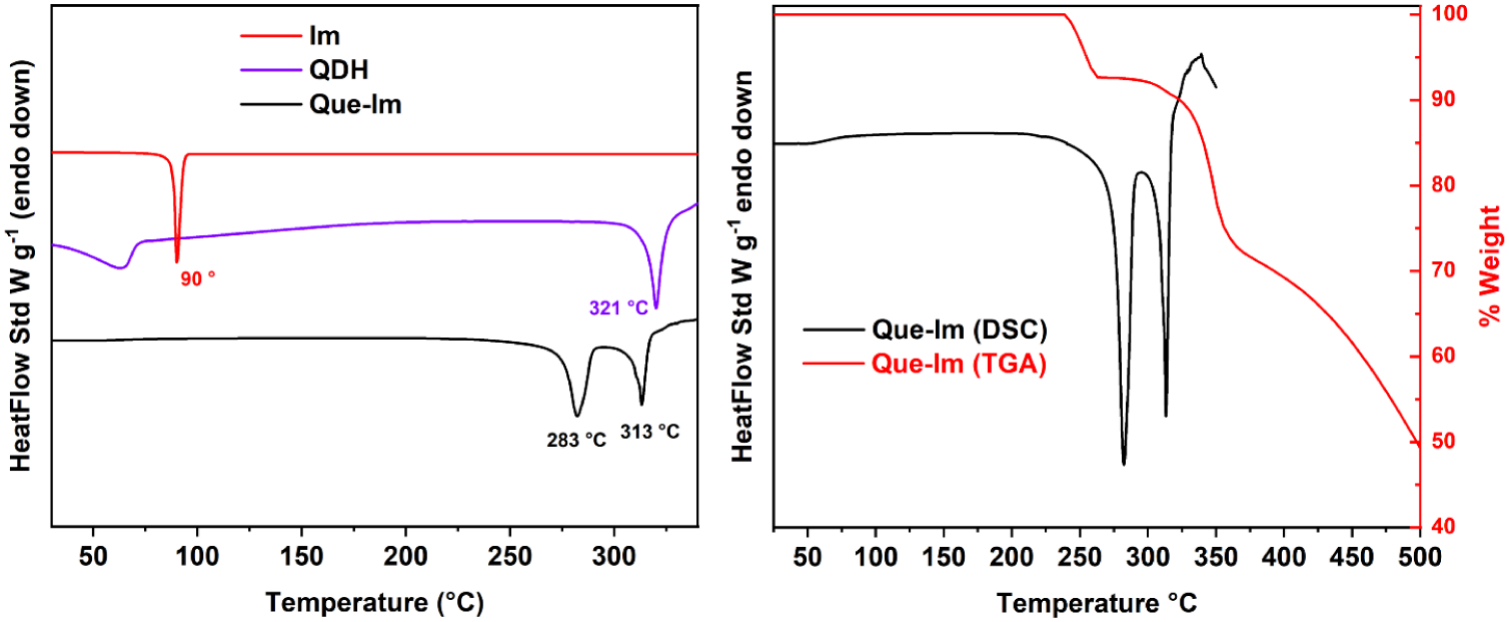
(Left) DSC comparisons of Que-Im (black), QDH (violet), and Im (red). (Right) TGA and DSC comparison of the Que-Im cocrystal. The thermal analysis was carried out under a N_2_ flux at 50 mL/min, with a heating rate of 10 °C/min.

The cocrystal shows a thermal behavior different from the starting materials and a degradation temperature lower than that of QDH. Que-Im exhibits two endothermic events, at 283 and 313 °C, which correspond to the weight loss steps in the TGA measurement. The first endothermic event (Figure 3, left) can be related to the loss of Im (theoretical 9.8%, experimental 8.45%), while the following one can be related to the degradation of QDH. Thus, the thermal stability of the cocrystal is nearly comparable to that of QDH. The thermal stability of the cocrystal is also confirmed by VT-PXRD, as reported in Figure S2. Up to 200 °C, the powder pattern is comparable to the measurements carried out at room temperature, except for the temperature effects that cause a shift of the peaks. Above that temperature, the powder starts to change and becomes less crystalline, with an increase of the amorphous content, as clearly evident in the region between 20 and 30 2θ°, until reaching decomposition at 300 °C. The remaining peaks are related to the aluminum oxide sample holder. In addition, the solubility of the cocrystal is lower but very close to that of QDH, as shown in Figure 4. This is due to the higher thermodynamic stability of the cocrystal than the starting materials in the IPA solvent. This increased stability can be explained by the formation of stronger intermolecular interactions within its crystal lattice (e.g., H-bond, van der Waals, and secondary bond interactions) compared to those of the starting materials, which will be discussed in the following sections.

**Figure 4:**
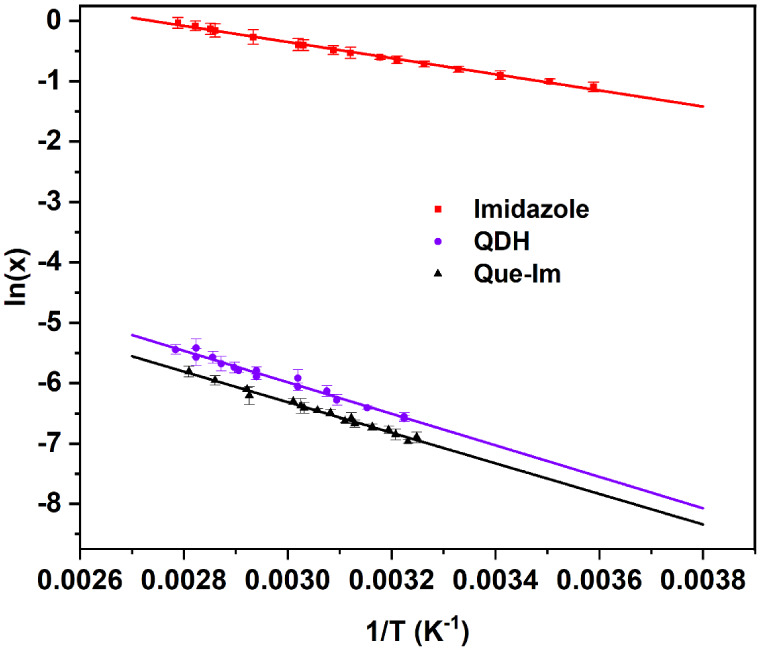
Solubility data of QDH, Que-Im, and imidazole collected in IPA. The van’t Hoff plots are represented by the lines, where *x* is the component molar fraction.

NMR spectroscopy provided further insights into the composition and nature of the Que-Im crystalline adduct. SSNMR analysis offers information about the purity, the number of independent molecules in the unit cell (*Z*′), and the degree of crystallinity of the samples under investigation. Furthermore, the chemical shifts of prominent resonances offer insights into the protonation state of ionizable groups and their contribution to H-bonds.^74,75^ Figure S3 reports the ^13^C CPMAS SSNMR spectrum of the cocrystal obtained, compared to those of the starting materials. All of the signal assignments are reported in Table S3 and are referred to the atom numbering shown in Figure 1.

A ^13^C NQS (non-quaternary suppression) experiment (Figure S4a), which allows an unequivocal assignment of the quaternary carbons, was acquired due to high peak overlapping in the region between 110 and 140 ppm. This analysis allowed us to unambiguously assign the C3′ and C3 signals (δ = 137.4 and 135.7 ppm, respectively). The C3 signal overlaps with the C2″H carbon of Im, as evidenced by the higher peak intensity in the ^13^C CPMAS spectrum compared to the ^13^C NQS one. Additionally, the C1’ signal falls at 124.3 ppm and overlaps with the aromatic carbon C4” or C5” of Im. By a careful evaluation of the ^13^C CPMAS spectrum of Que-Im (Figure S2), it was possible to distinguish the presence of a shoulder at 174.0 ppm associated with the C4 carbonyl signal (δ­(C=O) = 174.6 ppm). This shoulder, along with the large signal linewidth (full width at half-maximum at ∼130–220 Hz), indicates the presence of two independent molecules of Que in the cocrystal, in agreement with the nominal quantities used for the synthesis. The 2:1 stoichiometry was definitively confirmed via the integration of the signals in the solution ^1^H NMR spectrum, as shown in Figure S4b. The signal assignment is listed in Table S3. The integral values of 2 and 1 of the Im signals at 7.02 (H4" and H5") and 7.67 (H2") ppm, respectively, in relation to those of Que, each of which integrates 2, shows the presence of one molecule of Im and two molecules of Que in the adduct.

More detailed information about the ionic or neutral nature of the adduct was obtained through ^15^N CPMAS SSNMR experiments. In the ^15^N CPMAS spectrum of Im (Figure 5), two distinct signals can be observed at 174.2 and 245.5 ppm, assigned to N1” and N3”, respectively. The difference of 71.0 ppm between the two resonances is indicative of the formation of the H-bond N1”-H···N3”.^76^ In the ^15^N CPMAS spectrum of Que-Im, a shift toward lower frequencies of 9.2 ppm for N1’’ (δ = 165.0 ppm) and 18.1 ppm for N3′’ (δ = 227.4 ppm) is observed (Table S4). A similar behavior was reported in a niclosamide-imidazole cocrystal,^77^ where the shift to lower frequencies of both the Im peaks was attributed to the involvement of the *pyridine-like* nitrogen of Im as an acceptor in an H-bond interaction without proton transfer, e.g., O–H···N. This finding suggests the neutral nature of the Que-Im adduct.

**Figure 5:**
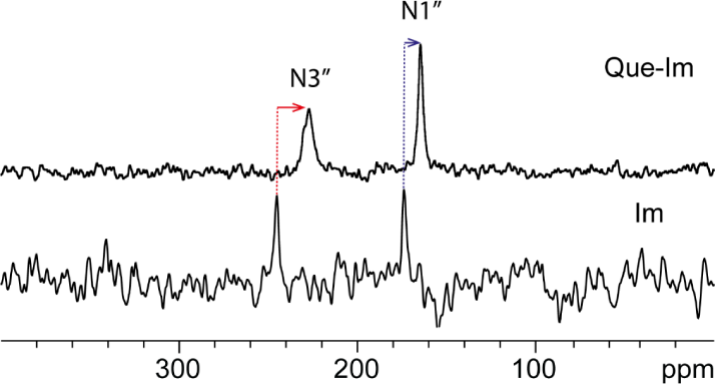
^15^N (40.56 MHz) CPMAS spectra of Que-Im and Im acquired with a spinning speed of 9 kHz at room temperature. The red and blue arrows highlight the shifts of the signals on passing from pure Im to Que-Im cocrystal.

### Supramolecular Features of the Que-Im Crystal Structure

The SSNMR measurements agree with the crystal structure of Que-Im, as determined by SXRD. Yellow plate crystals of Que-Im, suitable for SXRD analysis, were grown in the CrystalBreeder and are shown in Figure 6.

**Figure 6:**
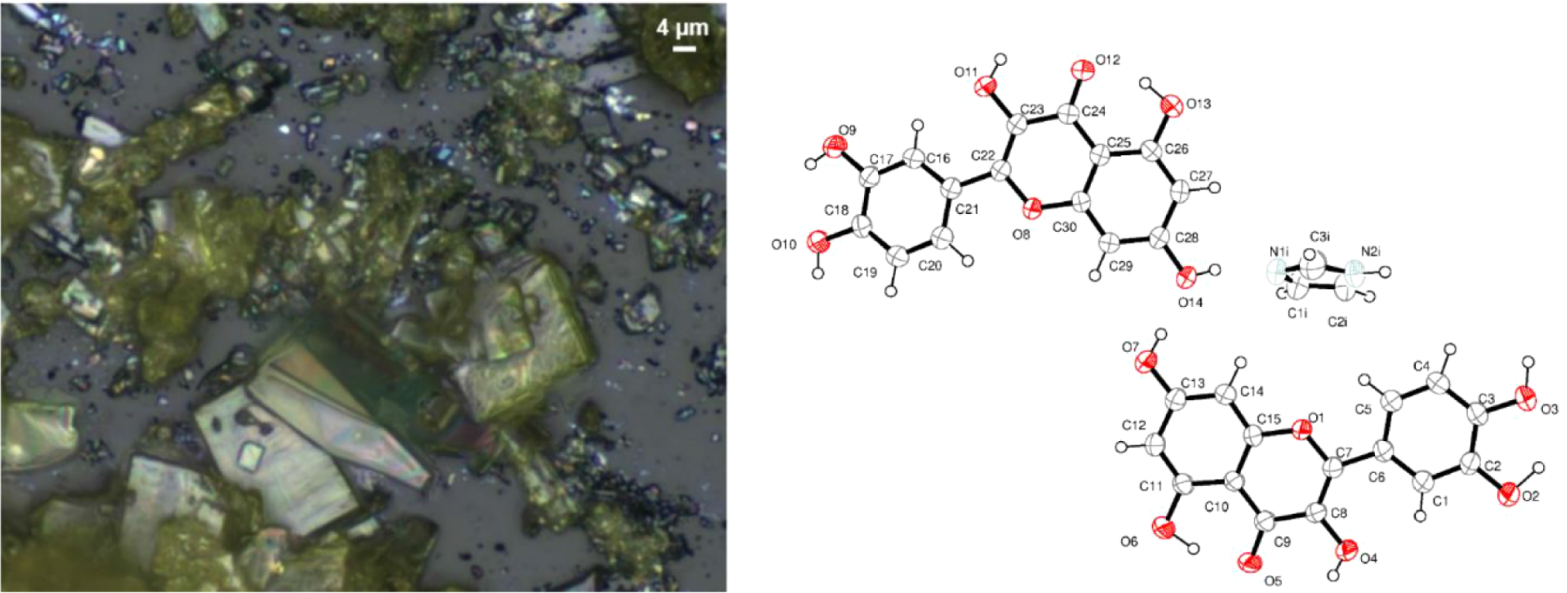
(Left) Optical microscope image of Que-Im crystallization. (Right) Molecular structure of Que-Im. Ellipsoids are drawn at 50% probability level.

Que-Im crystallizes in the triclinic space group *P*1̅ with two Que and one Im in the asymmetric unit (Figure 6). The full crystallographic data and refinement details are reported in Table S5. The phenyl and pyrone rings are almost coplanar, with dihedral angles of 4.71(4)° and 9.10(3)° around the C7–C6 and C16–C22 covalent bonds, respectively. These values are very close to those reported for known structures of Que hydrates and solvates, where the presence of a solvent molecule in the crystal structure leads to a planar conformation of the molecular backbone.^43–45^ The bond distances in the crystal structure are characteristic of conjugated aromatic systems and are reported in Tables S6 and S7. The molecules of Que and Im are arranged in infinite planar ribbons along the *a*-axis through the formation of strong H-bonds (in the range between 1.742 and 2.043 Å) between the O–H and N–H donor groups of Que and Im (Figure 7a). Thus, supramolecular heteroatomic interactions of Que and Im result in the formation of ring motifs along the aforementioned crystallographic ribbon. The two phenyl rings establish intramolecular interactions with the carboxylic groups of the pyrone rings, with characteristic bond lengths of 1.633(3) and 1.754(4) Å. The Que molecules are stacked along the *c*-axis with distances ranging from 3.355(8) to 3.399(3) Å (see Figure 7b).

**Figure 7:**
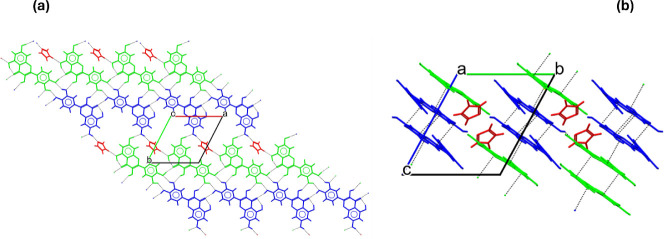
Crystal packing of Que-Im, viewed along the *c*-axis (a) and the *a*-axis (b). The molecules are colored by symmetry equivalence, and the H-bonding and π–π stacking interactions are drawn with black dashed lines.

The interaction energies of the supramolecular synthons and all of the unsaturated interactions that contribute to the attachment energy (AE) of the most morphologically important facets of the Que-Im crystal structure were calculated. In this way, the most important bulk intermolecular interactions in the crystal structure were ranked to understand how the interaction energies affect the crystal facet growth and their chemical nature. In Table 3, a summary of the most important intermolecular interactions is reported. Interaction distances, energies, and contributions to the total lattice energy were considered for the ranking.

**Table 3:** Intermolecular Interaction Energy of Que-Im

Interaction number	Molecules	Interaction type	Centroid–centroid distance (Å)	Interaction energy (kcal mol^–1^)	**%** to total lattice energy
synthon 1	Que1–Que2	H-bond	8.35	–8.42	21.3%
synthon 2	Que1–Que2	π–π stacking	3.56	–6.97	17.8%
synthon 3	Que1–Que1/Que2–Que2	offset stacking	6.15	–5.10	13%
synthon 4	Que1–Que2	H-bond	13.664	–4.64	11.8%
synthon 5	Que1-Im (donor)	H-bond	8.59	–3.18	8,1%
synthon 6	Que2-Im (acceptor)	H-bond	9.67	–2.88	7.3%
				total	79.3%

The six strongest synthons are represented in Figure 8, where the interactions are highlighted by dashed black lines. The contribution of the H-bonding in the ribbon of the Que molecules is found as the most energetic synthon, which is responsible for the planar conformation of the Que molecules in the crystal packing.

**Figure 8:**
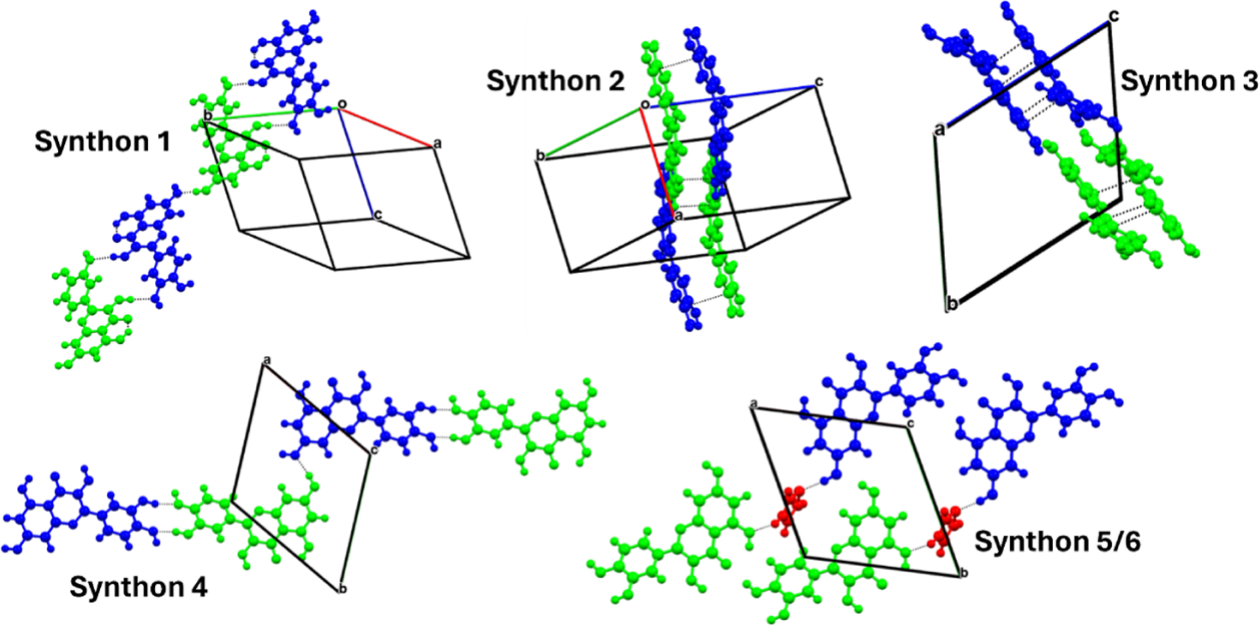
Principal synthons in Que-Im. Que1 (green), Que2 (blue), and Im (red). The interactions are highlighted with dashed black lines.

The planar conformation of the Que molecules in the structure promotes stacking interactions (π–π and offset) that are ranked as the second and third strongest synthons in the crystal lattice, with a very short distance of stacking, especially for synthon 2 (3.56 Å). The fourth most important synthon is the hydroxy chain $R_{2}^{2}\left(10\right)$ homomeric interaction, which contributes to the extension of the Que ribbon molecules along the *a*-axis. The Im molecules link, via H-bonds, the two independent Que molecules by acting as HBDs through the acidic N–H proton (synthon 5) and HBAs with the sp^2^ nitrogen atom (synthon 6). These six different synthons contribute to almost 80% of the total lattice energy, with a higher contribution from the H-bond interactions (48.5%) compared to other nonpolar interactions (30.8%). By comparing the energies of the calculated supramolecular synthons that contribute the most to the total lattice energy of the cocrystal with those calculated for the QDH and Que monohydrate structures reported in the CSD,^44^ it is possible to assess that the Que-Im cocrystal has the highest contribution from H-bond synthons (19.12 kcal mol^–1^) and π–π stacking interactions (12.07 kcal mol^–1^). In fact, these contributions are only 6.87 kcal mol^–1^ (H-bond) and 8.73 kcal mol^–1^ (π–π stacking) for QDH, and 13.52 kcal mol^–1^ (H-bond) and 6.80 kcal mol^–1^ (π–π stacking) for the monohydrate. These values indicate that the cocrystal presents greater strength of supramolecular interactions, which helps lower the energy of this crystal lattice, thus making the cocrystal more energetically stable than the two hydrated forms.

### Facet-Specific Surface Chemistry Analysis

Facet-specific surface properties are determined by the crystal structure: the calculation of topological features, surface terminations, and surface properties of the exposed functional groups on the facets can help in finding the exact relationship between the crystal structure and particle properties such as roughness and wettability (e.g., hydrophobicity, hydrophilicity). To characterize the surface chemistry and topological features of the Que-Im cocrystal, the predicted morphology obtained from the attachment energy model^78^ was analyzed and is shown in Figure 9.

**Figure 9:**
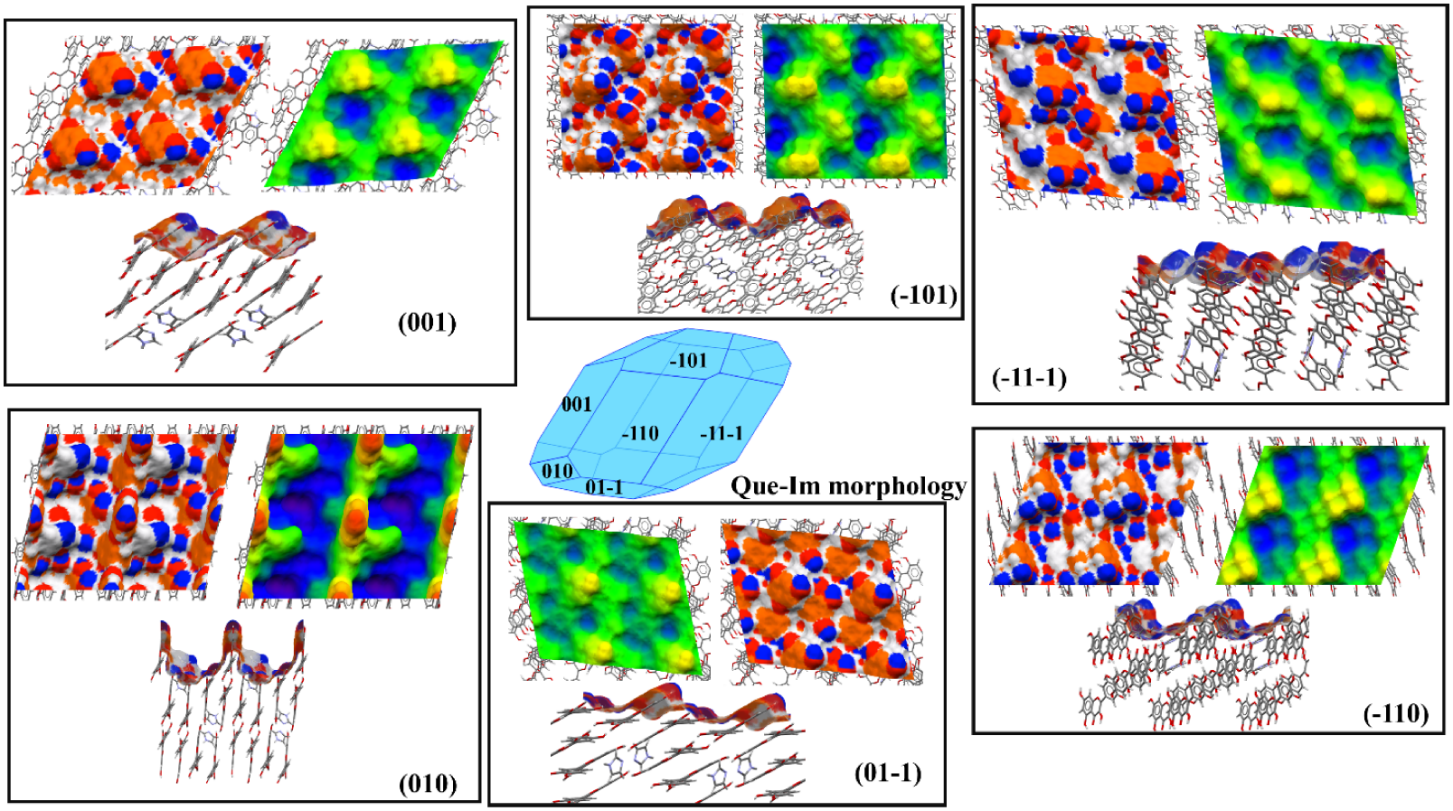
A 2 × 2 representation of the Que-Im facets. Surface topology and rugosity comparison for Que-Im: average plane (green), the region above the average plane (yellow and red), and the region below the average plane (blue). The projected areas of the six facets (001), (−101), (−11–1), (−110), (01–1), and (010) are 49, 59, 62, 61, 57, and 59 nm^2^. The terminations of the facets are shown in a side view for each facet. The atom properties are represented as HBDs (blue), HBAs (red), and aromatic bonds (orange).

Six prevalent facets were identified in the predicted morphology: {001}, {−101}, {−100}, {−11–1}, {−110}, and {010}. Three facets, {001}, {01–1}, and {1–11}, cover 69.28% of the total predicted crystal surface (26.22%, 23.66%, and 19.4% of the total area, respectively). Of these facets, the experimental ones determined by XRD, reported in Figures S5–S7 are the dominant {−101} and the lateral ones {−11–1} and {010}. All experimental facets are found in the predicted morphology with some differences in the relative areas; this is mostly because this estimation does not take into account the effect of the solvent on the crystal growth. The Que-Im facets {001}, {01–1}, and {1–11} grow mostly via stacking interactions and with a relatively low contribution of H-bonds. The offset stacking interaction between Que molecules (synthon 2) and the H-bonding interactions between Im and Que (synthons 5 and 6) contribute mostly to the attachment energy of the {001} facet, as shown in Table 4.

**Table 4:** Facet-Specific Properties of the Three Studied Solid Forms of Que^a^ ^,^ ^b^

Solid form	Aromatic bond density for the main facets (counts/Å^2^)	Attachment energy contribution of nonpolar interactions (kJ mol^–1^)	H-bond density for the main facets, donors (d), and acceptors (a) (counts/Å^2^)	Attachment energy contribution of polar interactions (kJ mol**^–1^)**
Que-Im	*d*_{–101}_ = 0.092	*E*_att{–101}_ = −22.68	*d*_{−101}_ = 0.074 (d), 0.062 (a)	*E*_att{–101}_ = −30.11
	*d*_{1–11}_ = 0.098	*E*_att{1–11}_ = −29.93	*d*_{1–11}_ = 0.052 (d), 0.064 (a)	*E*_att{1–11}_ = −26.62
	*d*_{010}_ = 0.126	*E*_att{010}_= −17.35	*d*_{010}_ = 0.084 (d), 0.105 (a)	*E*_att{010}–_= −46.66
QDH	*d*_{0–10}_ = 0.042	*E*_att{0–10}_ = −5.26	*d*_{0–10}_ = 0.083 (d), 0.104 (a)	*E*_att{0–10}_ = −3.96
	*d*_{100}_ = 0.032	*E*_att{100}_ = −5.34	*d*_{100}_ = 0.081 (d), 0.097 (a)	*E*_att{100}_ = −14.53
	*d*_{1–10}_ = 0.107	*E*_att{1–10}_ = −7.96	*d*_{1–10}_ = 0.089 (d), 0.124 (a)	*E*_att{1–10}_ = −10.02
	*d*_{00–1}_ = 0.124	*E*_att{00–1}_ = −33.18	*d*_{00–1}_ = 0.072 (d), 0.088 (a)	*E*_att{00–1}–_= −11.13
	*d*_{01–1}_ = 0.135	*E*_att{01–1}_ = −33.41	*d*_{01–1}_ = 0.073 (d), 0.094 (a)	*E*_att{01–1}_ = −13.97
QDMSO	*d*_{002}_ = 0.048	*E*_att{002}_ = −7.19	*d*_{002}_ = 0.035 (d), 0.044 (a)	*E*_att{002}_ = −8.50
	*d*_{0–11}_ = 0.083	*E*_att{0–11}_ = −8.53	*d*_{0–11}_ = 0.031 (d), 0.060 (a)	*E*_att{0–11}_ = −1.64
	*d*_{1–10}_ = 0.084	*E*_att{1–10}_= −16.50	*d*_{1–10}_ = 0.039 (d), 0.070 (a)	*E*_att{1–10}_ = −8.40

a Attachment energy contribution and bond density were calculated with mercury for both polar and nonpolar (van der Waals) interactions.

b Facets are ranked based on their relative areas in the experimental morphology, from the largest to the smallest.

This facet termination shows Im (yellow hills in the topology representation of Figure 9) and Que molecules tilted almost 45° with respect to the direction of growth of the facet, with their aromatic rings exposed (orange areas in Figure 9). The hydrophobicity of this facet is related to the exposure of these aromatic groups (as shown by the high density of aromatic bonds, orange regions of Figure 9), while the hydrophilic component is mostly related to the presence of the −OH groups, which can act as HBDs and HBAs (0.073 and 0.057 counts/Å^2^, blue and red regions in Figure 9). Based on these considerations, we can consider the {001} facet to be of mixed hydrophilic/hydrophobic nature, due to the coexistence of hydroxyl groups that are free to give H-bond interactions and aromatic rings that cover most of the surface. The {1–11} and {−101} are very similar in terms of topology, with the exposed −OH groups from the phenyl ring not involved in any interaction with Que and Im molecules. The nonpolar interaction synthons 2 and 3 contribute mostly to these facets’ growth, together with H-bonding between Im and Que (synthons 5/6). As shown in Table 4, the density of the aromatic bonds and HBA and HBD groups for this facet is very similar to the values calculated for facets {−101} and {1–11}, which are very similar in chemical nature. Both hydrophilic and hydrophobic regions are evident; the hydroxyl groups of the Que phenyl ring correspond to the most exposed regions on the topology map (yellow hills in Figure 9) and are unsatisfied HBD groups, while the aromatic contribution is mostly accounted for by the Im molecules and the Que pyrone rings placed at a lower height compared to the OH groups. The calculated energy contributions of H-bond and van der Waals interactions for {10–1} and {1–10} are also very similar, as shown in Table 4.

For the {1–10} facet, synthons 3 and 4 contribute mostly to the growth of this facet. In this case, the yellow hills in the topology map are related only to the hydroxyl groups of Que rings that contribute to the density of HBD and HBA groups on the surface (0.079 and 0.059 counts/Å^2^). The blue valleys are related to the Im molecules that are orthogonal with respect to the growth of the facet and contribute mostly to the density of aromatic bonds on the surface of this facet (0.093 counts/Å^2^). The {10–1} facet termination is characterized by Que molecules tilted by almost 30° with respect to the facet growth, with hydroxyl groups exposed (yellow hills).

Differently, the attachment energy of the {010} facet is dominated by the strongest H-bonding interaction found in the bulk synthon analysis (synthon 1), in combination with the H-bonding interactions between Im and Que (synthons 5 and 6). The amount of area occupied by the {010} facet is the lowest (7.54%), but it is characterized by the highest energetic contribution from H-bond interactions among all the analyzed facets. In fact, its growth occurs in the direction of the previously mentioned Que ribbons along the *a*-axis, where these ribbons are linked by strong H-bonds. This structural feature is also highlighted in the topology map of the {010} surface, which exhibits higher rugosity compared to the other two facets, with Que molecules orthogonal to the surface. The orthogonal orientation of the Que molecules also reduces the contribution of the nonpolar interactions to the surface energy of the {010} facet compared to the other two facets. By analyzing the surface map of the {010} facet, the contribution of the HBD and HBA groups is the highest among all facets (see Table 4), due to not only the exposure of the two hydroxyl groups of the Que phenyl ring but also of the N–H donor group of Im. Hence, the {010} facet appears to have a more polar and hydrophilic nature in comparison to the others, although both polar and nonpolar groups are exposed.

In summary, as shown in Table 4, the main facets of Que-Im are uniform, with similar attachment energy contributions (polar and nonpolar) and bond densities. This means that the surface properties of this solid form will not be significantly affected by the particle morphology.

In order to evaluate how the cocrystallization process has affected the surface properties of a solid form containing Que, we compared this structure with those of two known crystal forms of Que: QDH (Refcode FEFBEX01) and QDMSO (Refcode VUVHOM). The synthonic modeling and surface chemistry analysis performed by Klitou et al.^43^ highlight the anisotropy of these two solid forms, as indicated by the contribution of synthons of different natures (polar and nonpolar) to the attachment energy of each facet. Starting from these data, we have further studied facet-specific surface properties of these Que solid forms. In particular, the topology and the contributions of HBD, HBA, and nonpolar interactions were calculated for each facet (Figures 10 and 11).

**Figure 10:**
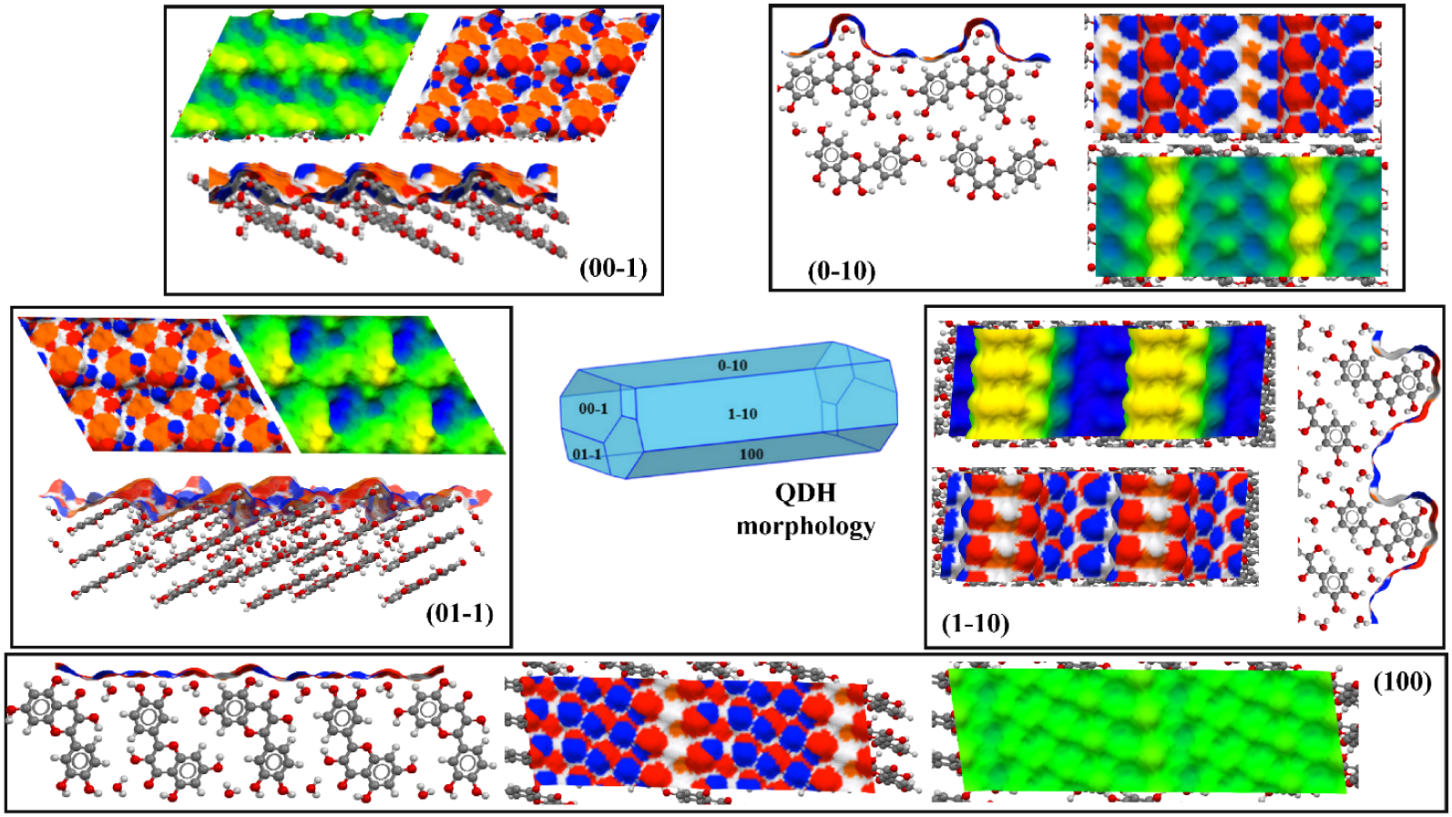
A 2 × 2 representation of the QDH facets. Surface topology and rugosity comparison for QDH: the average plane (green), region above the average plane (yellow), and region below the average plane (blue). The projected areas of the five facets (00–1), (01–1), (0–10), (100), and (1–10) are 78, 77, 50, 44, and 43 nm^2^.The terminations of the facets are shown in the top and side views for each facet. The atom properties are represented as HBDs (blue), HBAs (red), and aromatic bonds (orange).

**Figure 11:**
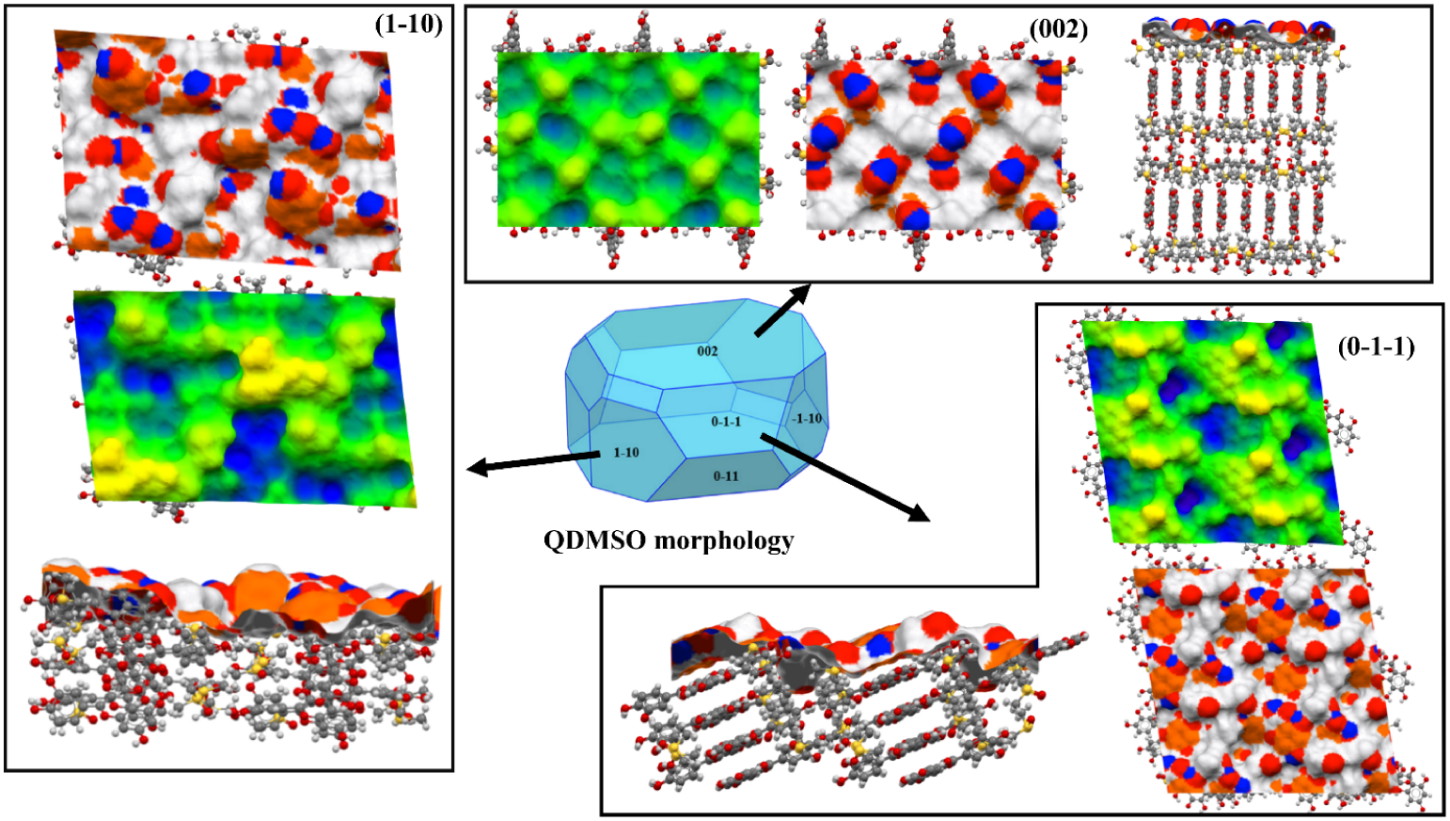
A 2 × 2 representation of the QDMSO facets. Surface topology and rugosity comparison for QDMSO: the average plane (green), the region above the average plane (yellow), and the region below the average plane (blue). The terminations of the facets are shown in the top and side views for each facet. The atom properties are represented as HBDs (blue), HBAs (red), and aromatic bonds (orange).

The QDH solid form shows clearly an anisotropic nature in terms of surface properties, as can be observed in Figure 10 and Table 4. The capping facets {00–1} and {01–1} present relatively smooth, hydrophobic surfaces, with Que molecules exposing mostly their aromatic rings, together with a hydroxyl group of a phenyl ring. On the other hand, the {0–10} and {1–10} facets are rougher and more hydrophilic, as shown in Figure 10. As shown in Table 4, for these facets, the contribution of HBDs and HBAs is much higher than that of the aromatic bonds. This is because, on these facets, water molecules and different hydroxyl groups are exposed. For the {0–10} facet, the yellow hills correspond to water molecules that have the capacity to be both HBDs and HBAs; while for the {1–10}, the roughness is due to the fact that the Que pyrone rings are exposed on the facet termination with two hydroxyl groups, one of which is involved in an intermolecular H-bond with the carboxyl group. Lastly, the {100} facet shows a smooth surface because in its direction of growth, water molecules are interlayered between molecular ribbons. In this case, the surface density of polar interactions is dominant, as clearly evident in Figure 10, where the blue and red areas cover most of the surface. Compared to Que-Im, QDH is more anisotropic in terms of surface properties; as shown in Table 4, facets {0–10} and {100} are dominated by polar groups, with minimal density of aromatic nonpolar bonds. Whereas, facets {00–1}, {01–1}, and {1–10} show the highest density of aromatic bonds of all three forms compared. This means that morphology will have a strong effect on surface chemistry, with the possibility to manipulate hydrophilicity/hydrophobicity by changing particle shape (e.g., using a different solvent or desupersaturation profile during manufacturing).

For the QDMSO crystal, the {002} facet represents the predicted most dominant one, with polar interactions that contribute slightly more than aromatic ones to the attachment energy.^45^ This feature is also evident from the topology map reported in Figure 11, in which the contribution of HBD and HBA groups is twice that of aromatic bonds. For the other two largest predicted facets, the {1–10} and {−110}, the density of aromatic bonds and the contribution of nonpolar interactions to these facets’ growth are dominant. Hence, the QDMSO is anisotropic in terms of surface properties, albeit less than the QDH.^43^

### Contact Angle Measurements

The surface analysis performed on the different solid forms was verified experimentally by contact angle measurements using water. SEM images of the three samples were collected before the analysis to check that the morphology was consistent with what was observed by SXRD measurements (Figure 12) and to verify that the crystal morphologies were needle-like or plate-like. As single crystals could not be obtained for facet-specific contact angle investigation, we used compacted powder disks to obtain an average measurement of the particle wettability with water. As shown in Figures 9 and 11, and by comparison with PXRD and SXRD measurements, the main contribution to the contact angle for QDMSO and the Que-Im cocrystal is given by the {002} and the {−101} facets, respectively. These solid forms present a plate-like morphology with the majority of the surface area occupied by these dominant facets. As also shown in previous literature,^43^ the QDH main facets obtained experimentally are the lateral {010} and {100}.

**Figure 12:**
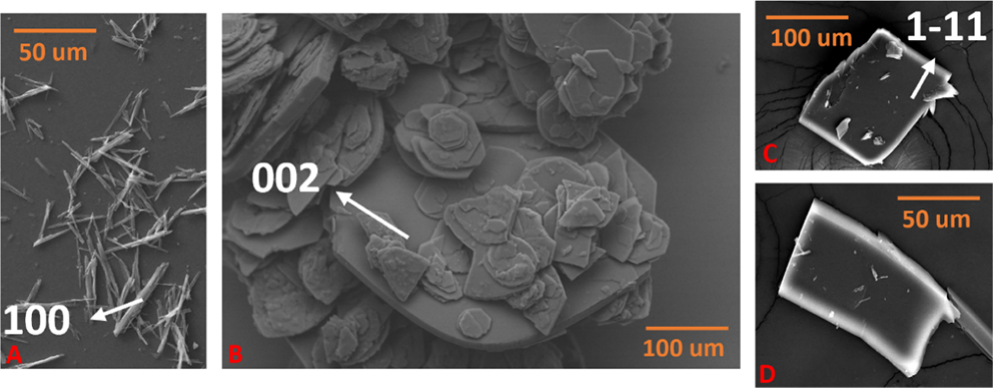
SEM images of (A) QDH, (B) QDMSO, and (C, D) Que-Im crystals. Facet orientations are depicted with white labels and arrows.

The results obtained from contact angle measurements are summarized in Table 5 and Figure 13.

**Figure 13:**
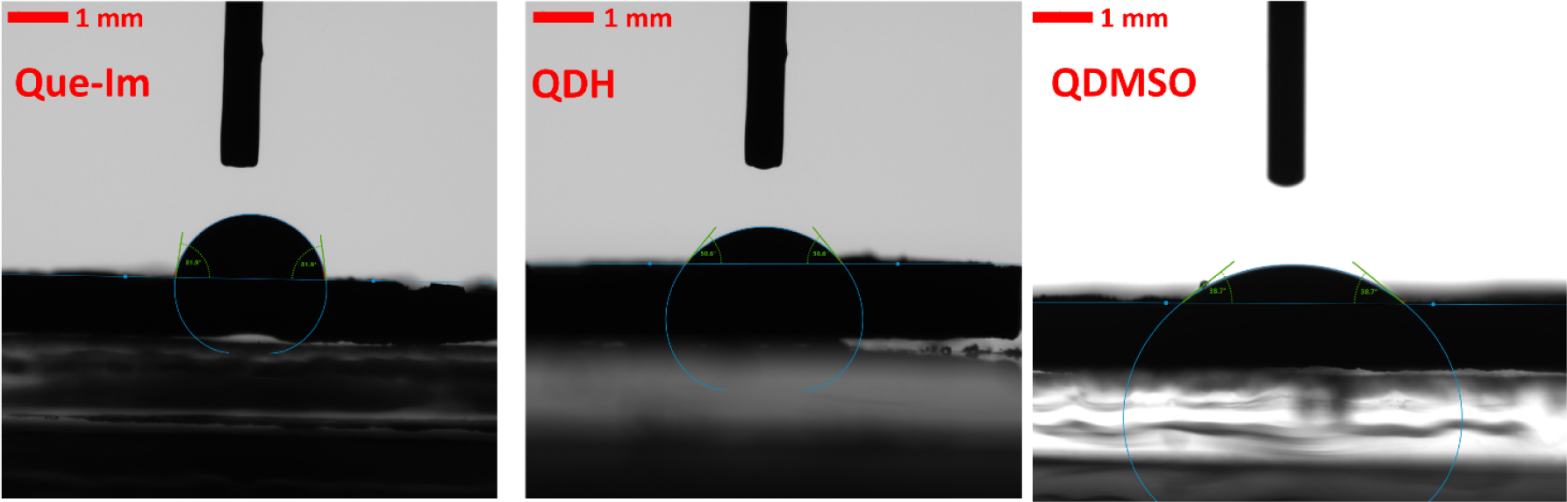
Contact angle measurements of Que-Im (left), QDH (center), and QDMSO (right).

**Table 5:** Contact Angle Values

Solid Form	Water
QDH	51.2 ± 5.2°
Que-Im	78.5 ± 3.9°
QDMSO	38.8 ± 1.0°

The results show that the Que-Im cocrystal is significantly more hydrophobic than the QDH and QDMSO (whose contact angle values are in accordance with previously measured ones).^45^ The QDMSO crystals, dominated in terms of morphology by the hydrophilic {002} facet, present a higher affinity with water. QDH crystals are also hydrophilic, but to a lesser degree compared to QDMSO. Despite having more hydrophilic facets, QDH is also the most anisotropic, with highly hydrophilic lateral facets and strongly hydrophobic capping facets that are present in the experimentally obtained crystals. Finally, Que-Im shows higher hydrophobicity, related to the absence of facets as hydrophilic as those found in QDMSO and QDH. The experimental data of contact angle measurements are in agreement with the modeling results, and the experimental PXRD measurements performed on the same disks (Figure S8) highlight the preferential orientation of Que-Im on the {1–11} facet, QDH on the {100} facet, and QDMSO on the {002} facet. The combination of experimental and computational analysis shows that Particle Informatics tools can reliably estimate surface properties, such as water wettability.

### Summary and Workflow Schematic

By combining experimental measurements and computational “Particle Informatics” tools, this work shows how cocrystallization can be used to tune the surface chemistry of particulate materials. Additionally, a practical workflow to apply cocrystallization as a method to manipulate the surface chemistry of particles was developed, as shown in Chart [Fig chart1]. The workflow starts with the selection of possible coformers and an in-silico screening using MC and MCHBP. A qualitative supramolecular analysis of the existing Que cocrystals was performed to rationally direct the selection of coformers for experimental screening. The coformers showing higher propensity for cocrystal formation are then screened experimentally (via slurrying), and a combination of PXRD, SSNMR, and Raman spectroscopy is used for unambiguous phase identification, based on chemical, structural, and thermal properties of the solid powder analyzed. After that, single cocrystals are grown by controlling temperature and solvent evaporation, and the crystal structure of the Que-Im cocrystal is determined via SXRD. Facet-specific surface properties of the solved structures can be calculated using Particle Informatics tools such as synthon analysis and surface and topology characterization. Most in-silico tools in the workflow make it a good option for the early stages of some product development (e.g., pharmaceuticals), when only small amounts of material are available for experiments and high-throughput screening and precise analytical surface characterization (e.g., atomic force microscopy) are not feasible.

## Conclusions

The presented work shows how crystal engineering, and cocrystallization in particular, can be used to modify facet-specific surface properties of organic crystalline materials. Through an in-silico study of facet-specific chemistry and topology, different crystal forms of Que were compared, and clear links between crystal structure and surface properties, such as hydrophilicity and hydrophobicity, were determined. The simulation results provided indications not only of the best Que structure for a specific application (e.g., hydrophobic particles required) but also of the best morphology to try and obtain via the crystallization process (e.g., maximizing specific crystallographic facets).

In particular, in this work, we developed a useful workflow, combining experimental and modeling tools, to apply cocrystallization as a crystal engineering strategy to control surface properties of particulate materials. Que was used as a model compound; it was found that cocrystallization with Im results in particles that are more uniform in terms of surface properties compared to the commercial dihydrate form, QDH, and a known solvate, QDMSO. In general, the Que-Im cocrystal exhibited greater hydrophobicity compared to the other two structures studied, and its surface properties were less dependent on the morphology (similar surface chemistry for all predicted facets). This can be a great advantage in terms of manufacturing, since factors that might affect morphology (e.g., crystallization solvent) would not dramatically impact the surface properties of particles, which are important for downstream operations’ efficiency.

The workflow starts with the use of in silico tools to identify suitable coformers. Based on the results of the in-silico screening, we conducted slurry crystallization experiments that helped in the isolation of a Que-Im cocrystal, which was characterized via XRD, Raman spectroscopy, SSNMR, and thermal analysis. Facet-specific surface chemistry and topology were calculated by using CCDC tools to compare the different crystal structures in terms of relative hydrophilicity and surface anisotropy. The Que-Im cocrystal was compared with known QDH and QDMSO structures; the cocrystal facets were found to be relatively uniform, with both nonpolar (aromatic) and polar regions. The QDH and QDMSO instead showed higher anisotropy, with some facets dominated by polar regions (e.g., able to accept and donate H-bonds) and others showing only the aromatic rings. The tendency of Que to arrange in planar ribbons within the crystal packings generated an overall crystal anisotropy, with hydrophilic facets growing in the crystallographic growth direction of the ribbons and hydrophobic facets growing perpendicularly to them. In terms of topology, it was found that a higher rugosity of the facets is related to a higher contribution of polar terminations on the surface, such as −OH groups, N–H of the Im moiety, water, and DMSO. Smoother facets tend to present a higher contribution of aromatic bonds on the surface, indicating a less hydrophilic nature. The workflow terminates with simulation validation, using contact angle measurements. These were conducted after careful analysis of the morphology of the measured particles, especially for the highly anisotropic QDH and QDMSO crystals. Experimental results showed good agreement with the simulations, indicating the reliability of the developed workflow.

## Supplementary Material



## Notes

Tables of the coformers tested in the MC and MCHBP analysis, additional experimental PXRD data related to the cocrystal screening, VT-PXRD of the Que-Im cocrystal phase, ^1^H NMR, ^13^C CPMAS, and NQS spectra of Que-Im, ^13^C CPMAS of QDH and Im with signal assignments, crystallographic data tables of the Que-Im crystal structure, experimental crystal indexing of the Que-Im cocrystal, surface and topology calculations on the experimental morphology of Que-Im with crystal habit parameters, geometry optimization data of Que-Im, and PXRD of the powder disks of Que-Im, QDMSO, and QDH (PDF)
