# Supplementary material for: A Combined Experimental and Modeling Workflow to Tune Surface Properties of Organic Materials via Cocrystallization
Source: Chem Mater. 2025 Jul 17;37(15):5593–608. doi: 10.1021/acs.chemmater.5c00634 (PMC12355690; doi:10.1021/acs.chemmater.5c00634)
Supplement: Supplementary file 1 [file cm5c00634_si_001.pdf]

A Combined Experimental and Modeling Workflow to Tune Surface Properties of Organic Materials via Cocrystallization

*Emmanuele Parisi[a], Giulia del Duca[a], Emilia Prandini[a], Silvia Fraterrigo Garofalo[a], Chiara Rosso[b], Michele Remo Chierotti[b], Elena Simone[a]**

a Department of Applied Science and Technology (DISAT), Politecnico di Torino, Corso Duca degli Abruzzi 24, I-10129, Torino, Italy

b Department of Chemistry and NIS Centre, University of Torino, Via P. Giuria 7, I-10125, Torino, Italy

*Email: elena.simone@polito.it

Supporting information section

1. **Virtual cocrystal screening**
2. **Powder X-ray diffraction phase check**
3. **Variable Temperature X-ray diffraction**
4. **SSNMR data**
5. **Crystallographic data**
6. **Experimental Crystal indexing**
7. **Simulated crystal morphology**
8. **Geometry optimization**
9. **Powder X-ray diffraction preferential orientation on powder disk**

1. Virtual cocrystal screening

Table S1 Coformer list for the virtual cocrystal screening

(+)-camphoric_acid	L-tryptophan	lactobionic_acid
(-)-camphorsulfonic_acid	L-tyrosine	lactose
1-hydroxyethylidene-1,1-diphosphonic_acid	N-ethylacetamide	maleic_acid
2-amino-5-methylbenzoic_acid	Vanillina	malic_acid

3-methylpyridine	acesulfame	malonic_acid
4-acetamidobenzoic_acid	acetic_acid	maltitol
4-aminobenzoic_acid	acetophenone_oxime	mannitol
4-hydroxybenzoic_acid	acetylenedicarboxylic_acid	methanesulfonic_acid
Acetamide	adipic_acid	methylparaben
Ferulic Acid	alitame	monobutyrin
D-alanine	apigenin	nicotinamide
D-glucuronic_acid	azelaic_acid	oxalic_acid
D-pantothenol	benzoic_acid	pamoic_acid
EDTA	biotin	phthalamide
iso-ferulic acid	caprolactam	pimelic_acid
Hydroquinone	capsaicin	piperazine
L-arginine	cholic_acid	propylparaben
L-aspartic_acid	citric_acid	pyrazine
L-aspartic_acid_z	ethylparaben	riboflavin
L-glutamic_acid	folic_acid	saccharin
L-glutamic_acid_z	fumaric_acid	sorbic_acid
L-glutamine	gentisic_acid	suberic_acid
L-glutathione	glutaric_acid	succinic_acid
L-lactic_acid	glycine	t-butylamine
L-leucine	glycolic_acid	t-butylhydroxyanisole
L-mandelic_acid	hesperetin	theophylline
L-methionine	hippuric_acid	thymidine
L-phenylalanine	hydrocinnamic_acid	triphenylacetic_acid
L-proline	imidazole	urea
L-serine	isonicotinamide	valerolactam
L-tartaric_acid	ketoglutaric_acid	xanthine

The parameters threshold for PASS output are the following: M/L axis ratio D < 0.31, S axis (Å) < 3.23, S/L axis ratio D < 0.275, dipole moment magnitude (Debye) < 5.94, fraction of Nitrogen and Oxygen D < 0.294. D is the difference between the parameters values of the quercetin compared with each coformer reported in Table S2.

Table S2 Molecular complementarity screening global results.

Active Conformation	M/L axis ratio	S axis (Å)	S/L axis ratio	dipole moment magnitude (Debye)	fraction of Nitrogen and Oxygen	Overall result
**Quercetin**	0.682	4.671	0.331	5.328	0.318	
Coformer Conformation	M/L axis ratio	S axis (Å)	S/L axis ratio	dipole moment magnitude (Debye)	fraction of Nitrogen and Oxygen	
(+)-camphoric_acid	0.713	7.052	0.687	0.558	0.286	FAIL
(-)-camphorsulfonic_acid	0.827	7.313	0.738	3.432	0.267	FAIL
1-hydroxyethylidene-1.1-diphosphonic_acid	0.819	6.102	0.715	4.143	0.636	FAIL

2-amino-5-methylbenzoic_acid	0.768	4.169	0.408	1.361	0.273	PASS
3-methylpyridine	0.814	4.167	0.506	1.437	0.143	PASS
4-acetamidobenzoic_acid	0.536	4.174	0.32	1.612	0.308	PASS
4-aminobenzoic_acid	0.671	3.4	0.341	0.849	0.3	PASS
4-hydroxybenzoic_acid	0.675	3.4	0.343	2.205	0.3	PASS
Acetamide	0.944	3.969	0.636	1.804	0.5	FAIL
Ferulic Acid	0.62	3.514	0.31	0.687	0.182	PASS
D-alanine	0.942	5.087	0.764	3.343	0.5	FAIL
D-glucuronic_acid	0.774	6.475	0.676	3.657	0.538	FAIL
D-pantothenol	0.582	6.952	0.564	4.265	0.357	PASS
EDTA	0.68	6.615	0.487	3.05	0.5	PASS
iso-ferulic acid	0.714	4.207	0.341	3.307	0.286	PASS
Hydroquinone	0.77	3.415	0.394	0	0.25	PASS
L-arginine	0.576	5.881	0.463	2.027	0.5	PASS
L-aspartic_acid	0.891	5.364	0.655	1.186	0.556	FAIL
L-aspartic_acid_z	0.787	5.393	0.62	15.846	0.556	FAIL
L-glutamic_acid	0.743	5.521	0.563	1.697	0.5	PASS
L-glutamic_acid_z	0.701	5.556	0.564	15.835	0.5	FAIL
L-glutamine	0.775	5.887	0.658	0.564	0.5	FAIL
L-glutathione	0.839	6.855	0.478	0.646	0.45	PASS
L-lactic_acid	0.895	5.399	0.772	2.587	0.5	FAIL
L-leucine	0.851	5.688	0.654	3.422	0.333	FAIL
L-mandelic_acid	0.605	5.898	0.599	2.816	0.273	PASS
L-methionine	0.681	5.479	0.533	2.085	0.333	PASS
L-phenylalanine	0.577	5.457	0.473	1.37	0.25	PASS
L-proline	0.781	5.744	0.714	1.545	0.375	FAIL
L-serine	0.922	5.064	0.689	4.362	0.571	FAIL
L-tartaric_acid	0.961	5.335	0.669	3.295	0.6	FAIL
L-tryptophan	0.689	6.018	0.53	0.932	0.267	PASS
L-tyrosine	0.668	6.27	0.571	1.737	0.308	PASS
N-ethylacetamide	0.685	4.169	0.478	1.656	0.333	PASS
Vanillina	0.614	8.671	0.53	9.992	0.273	FAIL
acesulfame	0.998	5.177	0.636	2.386	0.5	FAIL
acetic_acid	0.882	4.175	0.699	0.86	0.5	FAIL
acetophenone_oxime	0.77	4.181	0.442	1.223	0.2	PASS
acetylenedicarboxylic_acid	0.654	3.777	0.418	0.428	0.5	PASS
adipic_acid	0.476	4.161	0.36	0.013	0.4	PASS
alitame	0.656	7.989	0.61	1.98	0.318	FAIL
apigenin	0.64	3.409	0.24	0.493	0.25	PASS
azelaic_acid	0.351	4.167	0.261	0.928	0.308	FAIL
benzoic_acid	0.723	3.401	0.367	0.991	0.222	PASS
biotin	0.659	6.324	0.471	1.638	0.313	PASS
caprolactam	0.95	5.535	0.696	2.061	0.25	FAIL
capsaicin	0.446	6.494	0.319	2.166	0.182	PASS
cholic_acid	0.453	7.399	0.404	4.539	0.172	PASS
citric_acid	0.673	6.273	0.618	1.783	0.538	FAIL
ethylparaben	0.637	4.371	0.383	3.594	0.25	PASS
folic_acid	0.421	8.053	0.367	2.021	0.406	FAIL
fumaric_acid	0.617	3.4	0.366	2.637	0.5	PASS
gentisic_acid	0.866	3.408	0.381	2.843	0.364	PASS
glutaric_acid	0.528	4.167	0.386	2.449	0.444	PASS
glycine	0.747	4.254	0.563	1.007	0.6	PASS
glycolic_acid	0.787	4.182	0.597	1.422	0.6	PASS

hesperetin	0.683	7.492	0.575	5.599	0.273	PASS
hippuric_acid	0.578	5.886	0.491	2.176	0.308	PASS
hydrocinnamic_acid	0.805	6.052	0.673	0.852	0.182	FAIL
imidazole	0.925	3.4	0.53	1.319	0.4	PASS
isonicotinamide	0.782	3.411	0.401	1.272	0.333	PASS
ketoglutaric_acid	0.577	4.171	0.389	1.37	0.5	PASS
lactobionic_acid	0.704	8.159	0.66	8.72	0.5	FAIL
lactose	0.687	6.591	0.515	4.554	0.478	PASS
maleic_acid	0.689	3.435	0.393	4.208	0.5	PASS
malic_acid	0.653	4.874	0.52	3.291	0.556	PASS
malonic_acid	0.694	4.15	0.527	1.699	0.571	PASS
maltitol	0.845	8.076	0.68	7.131	0.478	FAIL
mannitol	0.857	6.684	0.75	4.66	0.5	FAIL
methanesulfonic_acid	0.993	5.301	0.947	2.599	0.6	FAIL
methylparaben	0.666	4.23	0.402	1.291	0.273	PASS
monobutyrin	0.731	5.181	0.487	3.488	0.364	PASS
nicotinamide	0.724	3.408	0.371	2.435	0.333	PASS
oxalic_acid	0.845	3.4	0.498	2.623	0.667	FAIL
pamoic_acid	0.728	7.963	0.602	0.233	0.207	FAIL
phthalamide	0.955	4.943	0.558	2.875	0.333	PASS
pimelic_acid	0.431	4.18	0.322	1.664	0.364	PASS
piperazine	0.92	4.979	0.697	0.001	0.333	FAIL
propylparaben	0.532	4.913	0.371	2.286	0.231	PASS
pyrazine	0.904	3.41	0.525	0	0.333	PASS
riboflavin	0.833	6.995	0.502	3.978	0.37	PASS
saccharin	0.929	5.617	0.632	2.141	0.333	FAIL
sorbic_acid	0.502	4.166	0.38	1.028	0.25	PASS
suberic_acid	0.392	4.161	0.294	0.01	0.333	PASS
succinic_acid	0.584	4.189	0.443	1.076	0.5	PASS
t-butylamine	0.914	5.954	0.886	0.771	0.2	FAIL
t-butylhydroxyanisole	0.736	6.735	0.606	0.688	0.154	FAIL
theophylline	0.879	4.194	0.425	0.736	0.462	PASS
thymidine	0.7	7.542	0.646	4.421	0.412	FAIL
triphenylacetic_acid	0.967	8.343	0.742	3.073	0.091	FAIL
urea	0.869	3.4	0.525	1.44	0.75	FAIL
valerolactam	0.849	5.134	0.65	2.028	0.286	FAIL
xanthine	0.824	3.405	0.368	1.202	0.545	PASS

2. Powder X-ray diffraction phase check

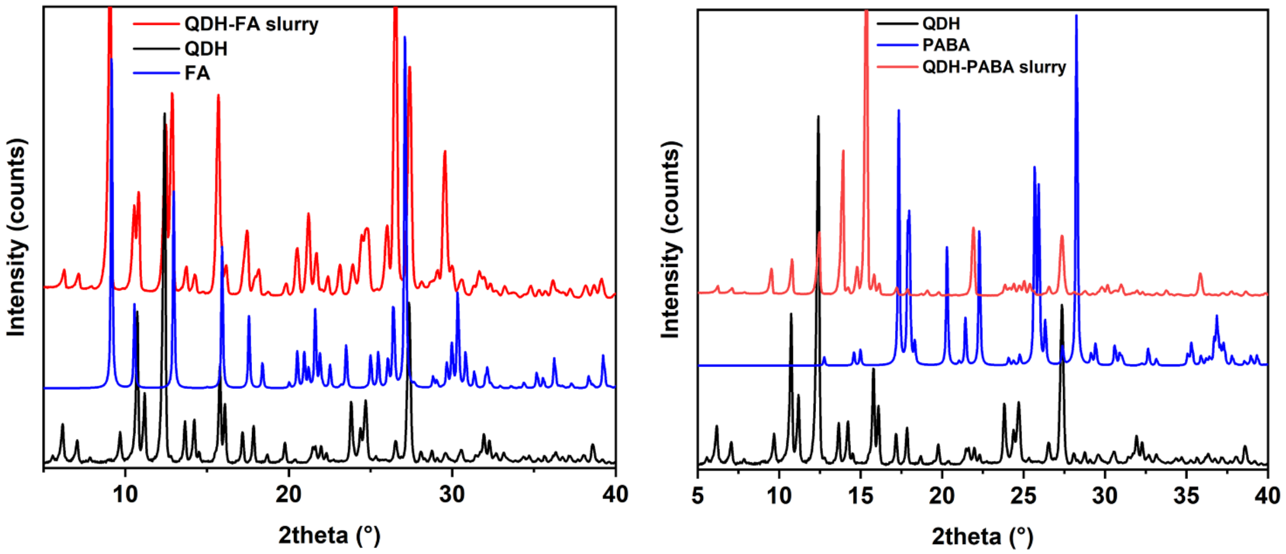

Figure S1 Comparison PXRD data QDH-FA (left) and QDH-PABA (Right) with starting material.

3. Variable-Temperature powder X-ray diffraction

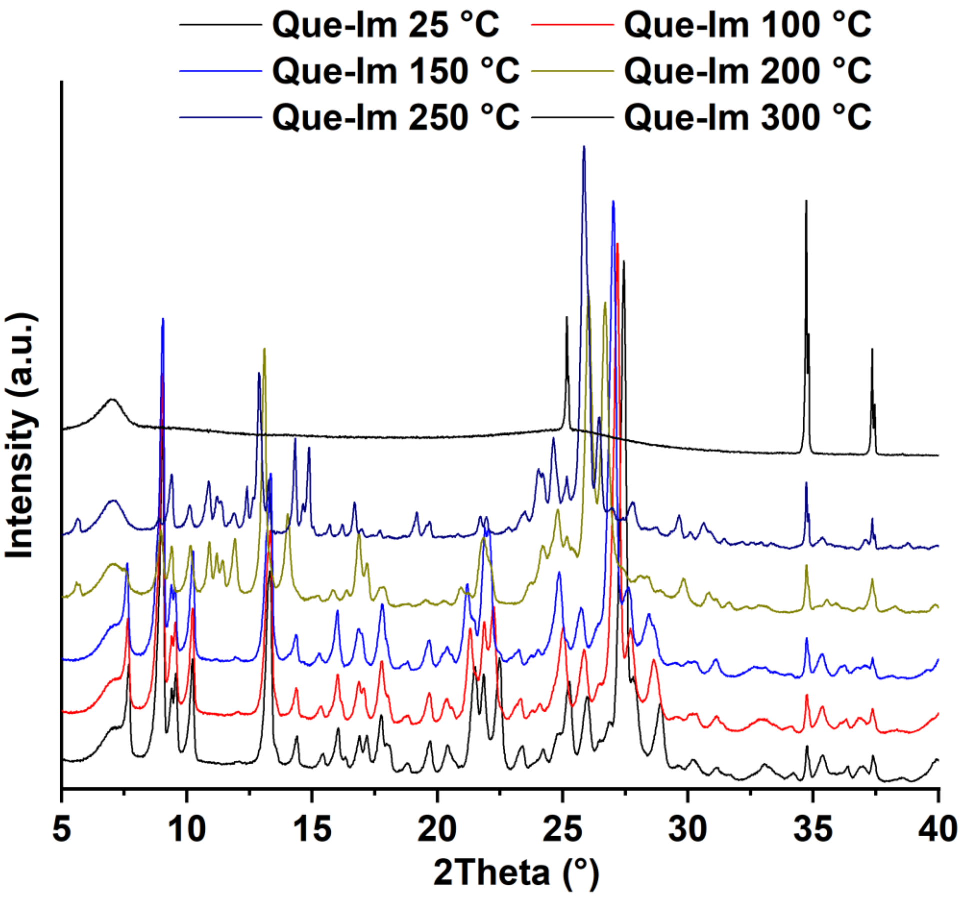

Figure S2 Variable-temperature powder-X-ray diffraction analysis of Que-Im cocrystal at different temperatures.

4. SSNMR data

Table S3 Signal assignment of ^{1}H-NMR spectrum in solution of Que-Im cocrystal in the region between 6.0 and 8.0 ppm.

Quercetin		Imidazole	
**Signal**	**d (ppm)**	**Signal**	**d (ppm)**
H2’	7.67	H2”	7.65
H6’	7.53	H4”/H5”	7.02
H5’	6.88		
H8	6.40		
H6	6.18		

Table S4 Signal assignment of ^{15}N-CPMAS SSNMR of imidazole and Que-Im cocrystal.

	Imidazole	Que-Im
**Signal**	**d (ppm)**	**d (ppm)**
N1”	174.2	165.0
N3”	245.2	227.4

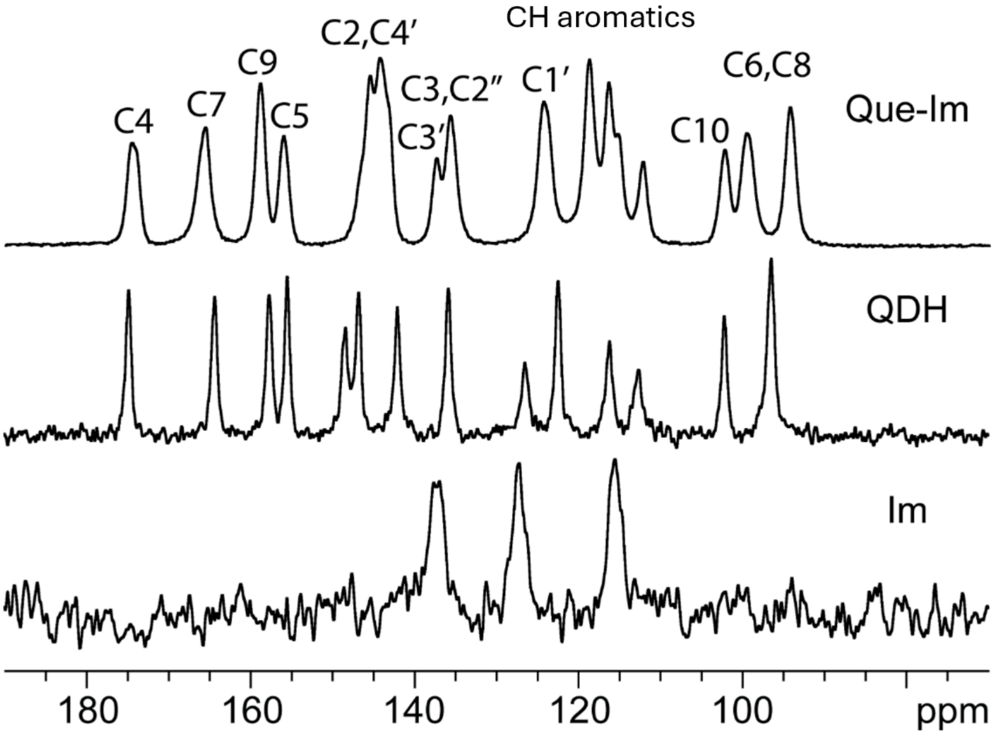

Figure S3 ^{13}C (100.63 MHz) CPMAS spectra of Que-Im (top), QDH (middle), and Im (bottom) acquired with a spinning speed of 12 kHz at room temperature.

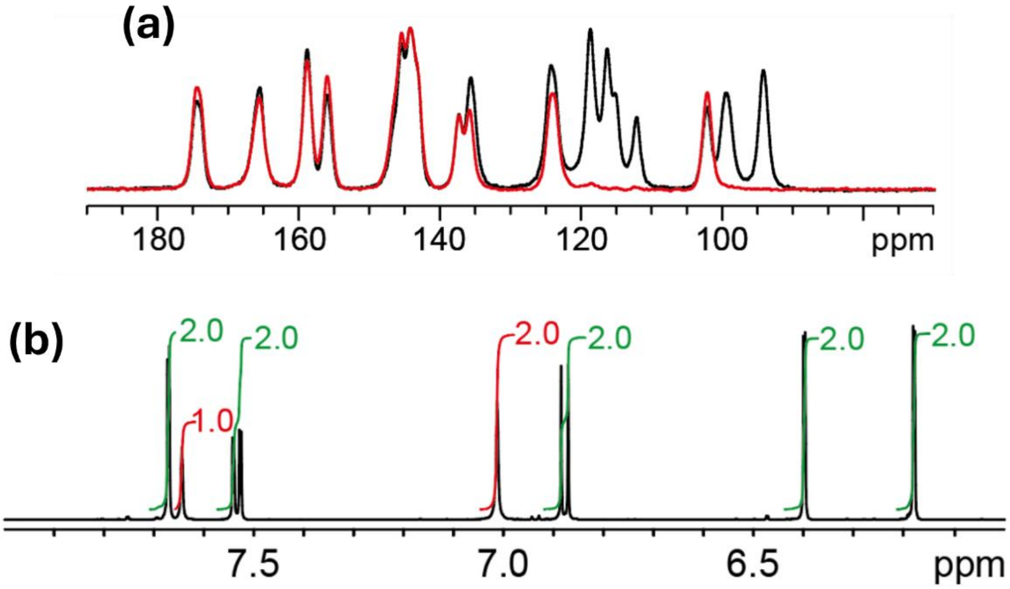

Figure S4 (a) ^{13}C (100.63 MHz) CPMAS (black) and NQS (red) spectra of Que-Im acquired with a spinning speed of 12 kHz at room temperature. (b) ^{1}H NMR spectrum (600.17 MHz) of Que-Im in DMSO-d6 at 25°C. In green are shown the signal integrations of Que, in red the ones of Im.

5. Crystallographic data

Table S5 Crystallographic experimental details of Que-Im data collection.

	Quercetin-Imidazole (Que-Im)
Crystal data	
Chemical formula	$2(C_{15}H_{10}O_7)\cdot C_3H_4N_2$
M_r	672.54
Crystal system. space group	Triclinic. $P\bar{1}$
Temperature (K)	298
a. *b*. *c* (Å)	10.9581 (8). 12.1229 (10). 13.0804 (12)
α, β, γ (°)	114.659 (8). 93.308 (7). 113.045 (7)
V (Å^3)	1401.5 (2)
Z	2
Radiation type	Cu *K*a
m (mm^{-1})	1.08
Crystal size (mm)	0.5 × 0.25 × 0.1
Data collection	
Diffractometer	Xcalibur. AtlasS2. Gemini ultra
Absorption correction	Multi-scan<br>*CrysAlis PRO* 1.171.42.71a (Rigaku Oxford Diffraction. 2022) Empirical

	absorption correction using spherical harmonics. implemented in SCALE3 ABSPACK scaling algorithm.
T_{min}. T_{max}	0.957. 1.000
No. of measured. independent and observed [$I > 2ó(I)$] reflections	16949. 4941. 3160
R_{int}	0.048
(sin q/l)$_{max}$ (Å^{-1})	0.597
Refinement	
$R[F^2 > 2s(F^2)]$. $wR(F^2)$. S	0.050. 0.146. 1.01
No. of reflections	4941
No. of parameters	482
H-atom treatment	H atoms treated by a mixture of independent and constrained refinement
Dr$_{max}$. Dr$_{min}$ (e Å^{-3})	0.27. -0.23

Computer programs: *CrysAlis PRO* 1.171.42.71a (Rigaku OD. 2022). *SHELXT* 2018/2 (Sheldrick. 2018). *SHELXL* 2016/6 (Sheldrick. 2015). Olex2 1.5-ac6-020 (Dolomanov *et al.*. 2009).

Table S6. Selected geometric parameters (º)

C15—O1—C7	122.11 (18)	C17—C16—C21	120.2 (2)
C2—O2—H2	103 (2)	C21—C16—H16	119.9 (2)
C3—O3—H3	111 (2)	O9—C17—C16	118.5 (2)
C8—O4—H4	113 (2)	O9—C17—C18	120.6 (2)
C11—O6—H6	102.4 (17)	C16—C17—C18	120.8 (2)
C13—O7—H7	112 (2)	O10—C18—C17	117.1 (2)
C2—C1—H1	119.9 (2)	O10—C18—C19	123.7 (2)
C6—C1—H1	119.9 (2)	C19—C18—C17	119.2 (2)
C6—C1—C2	120.1 (2)	C18—C19—H19	119.8 (2)
O2—C2—C1	118.8 (2)	C18—C19—C20	120.3 (2)
O2—C2—C3	120.8 (2)	C20—C19—H19	119.8 (2)
C3—C2—C1	120.4 (2)	C19—C20—H20	119.5 (2)
C2—C3—O3	116.5 (2)	C19—C20—C21	121.1 (2)
C4—C3—O3	123.6 (2)	C21—C20—H20	119.5 (2)
C4—C3—C2	119.9 (2)	C16—C21—C22	122.1 (2)
C3—C4—H4A	119.9 (2)	C20—C21—C16	118.3 (2)
C3—C4—C5	120.3 (2)	C20—C21—C22	119.4 (2)
C5—C4—H4A	119.9 (2)	O8—C22—C21	110.02 (19)
C4—C5—H5	119.4 (2)	C23—C22—O8	120.1 (2)
C4—C5—C6	121.2 (2)	C23—C22—C21	129.8 (2)

C6—C5—H5	119.4 (2)	O11—C23—C24	118.0 (2)
C1—C6—C7	123.2 (2)	C22—C23—O11	120.3 (2)
C5—C6—C1	118.1 (2)	C22—C23—C24	121.8 (2)
C5—C6—C7	118.7 (2)	O12—C24—C23	120.8 (2)
O1—C7—C6	110.14 (19)	O12—C24—C25	123.2 (2)
C8—C7—O1	119.96 (19)	C25—C24—C23	116.0 (2)
C8—C7—C6	129.9 (2)	C26—C25—C24	123.1 (2)
O4—C8—C9	118.4 (2)	C30—C25—C24	120.1 (2)
C7—C8—O4	120.4 (2)	C30—C25—C26	116.8 (2)
C7—C8—C9	121.2 (2)	O13—C26—C25	119.4 (2)
O5—C9—C8	120.6 (2)	O13—C26—C27	119.6 (2)
O5—C9—C10	123.0 (2)	C27—C26—C25	121.0 (2)
C10—C9—C8	116.5 (2)	C26—C27—H27	119.8 (2)
C9—C10—C11	123.2 (2)	C26—C27—C28	120.3 (2)
C15—C10—C9	120.2 (2)	C28—C27—H27	119.8 (2)
C15—C10—C11	116.6 (2)	O14—C28—C27	121.3 (2)
O6—C11—C10	118.4 (2)	O14—C28—C29	118.5 (2)
O6—C11—C12	120.6 (2)	C29—C28—C27	120.2 (2)
C12—C11—C10	121.0 (2)	C28—C29—H29	120.8 (2)
C11—C12—H12	119.9 (2)	C30—C29—C28	118.4 (2)
C11—C12—C13	120.1 (2)	C30—C29—H29	120.8 (2)
C13—C12—H12	119.9 (2)	O8—C30—C25	120.4 (2)
O7—C13—C12	116.7 (2)	O8—C30—C29	116.3 (2)
O7—C13—C14	122.3 (2)	C29—C30—C25	123.3 (2)
C14—C13—C12	121.0 (2)	C3I—N1I—C1I	105.6 (3)
C13—C14—H14	121.2 (2)	C2I—N2I—H2I	132.0 (3)
C15—C14—C13	117.7 (2)	C3I—N2I—H2I	119.0 (3)
C15—C14—H14	121.2 (2)	C3I—N2I—C2I	108.4 (3)
O1—C15—C10	120.0 (2)	N1I—C1I—H1I	129.0 (2)
O1—C15—C14	116.4 (2)	C2I—C1I—N1I	109.8 (4)
C14—C15—C10	123.6 (2)	C2I—C1I—H1I	121.0 (2)
C30—O8—C22	121.64 (18)	N2I—C2I—H2IA	126.0 (2)
C17—O9—H9	107.0 (2)	C1I—C2I—N2I	105.9 (4)
C18—O10—H10	112.0 (2)	C1I—C2I—H2IA	128.0 (2)
C23—O11—H11	111.0 (2)	N1I—C3I—N2I	110.3 (4)
C26—O13—H13	107.0 (2)	N1I—C3I—H3I	123.0 (2)
C28—O14—H14A	109.5 (2)	N2I—C3I—H3I	126.0 (2)
C17—C16—H16	119.9 (2)		

Table S7. Selected geometric parameters (Å)

O1—C7	1.364 (3)	O10—C18	1.365 (3)
O1—C15	1.361 (3)	O11—H11	0.82 (4)
O2—H2	0.98 (4)	O11—C23	1.359 (3)
O2—C2	1.355 (3)	O12—C24	1.257 (3)
O3—H3	0.91 (3)	O13—H13	0.91 (3)
O3—C3	1.378 (3)	O13—C26	1.355 (3)
O4—H4	0.82 (3)	O14—H14A	0.8200
O4—C8	1.361 (3)	O14—C28	1.333 (3)
O5—C9	1.263 (3)	C16—H16	0.9300
O6—H6	0.96 (3)	C16—C17	1.377 (3)
O6—C11	1.357 (3)	C16—C21	1.399 (3)
O7—H7	0.87 (4)	C17—C18	1.387 (4)
O7—C13	1.341 (3)	C18—C19	1.378 (4)
C1—H1	0.9300	C19—H19	0.9300
C1—C2	1.390 (3)	C19—C20	1.380 (4)
C1—C6	1.389 (3)	C20—H20	0.9300
C2—C3	1.375 (4)	C20—C21	1.388 (4)
C3—C4	1.361 (3)	C21—C22	1.474 (3)
C4—H4A	0.9300	C22—C23	1.351 (3)
C4—C5	1.380 (3)	C23—C24	1.445 (3)
C5—H5	0.9300	C24—C25	1.433 (3)
C5—C6	1.388 (3)	C25—C26	1.416 (3)
C6—C7	1.473 (3)	C25—C30	1.391 (3)
C7—C8	1.358 (3)	C26—C27	1.365 (3)
C8—C9	1.441 (3)	C27—H27	0.9300
C9—C10	1.423 (3)	C27—C28	1.399 (3)
C10—C11	1.424 (3)	C28—C29	1.389 (3)
C10—C15	1.391 (3)	C29—H29	0.9300
C11—C12	1.360 (3)	C29—C30	1.376 (3)
C12—H12	0.9300	N1I—C1I	1.377 (5)
C12—C13	1.396 (3)	N1I—C3I	1.310 (4)
C13—C14	1.388 (3)	N2I—H2I	0.93 (4)
C14—H14	0.9300	N2I—C2I	1.357 (5)
C14—C15	1.381 (3)	N2I—C3I	1.329 (5)
O8—C22	1.371 (3)	C1I—H1I	1.02 (3)
O8—C30	1.364 (3)	C1I—C2I	1.328 (4)
O9—H9	0.86 (4)	C2I—H2IA	0.98 (4)
O9—C17	1.365 (3)	C3I—H3I	0.97 (4)
O10—H10	0.83 (4)		

6. Experimental crystal indexing

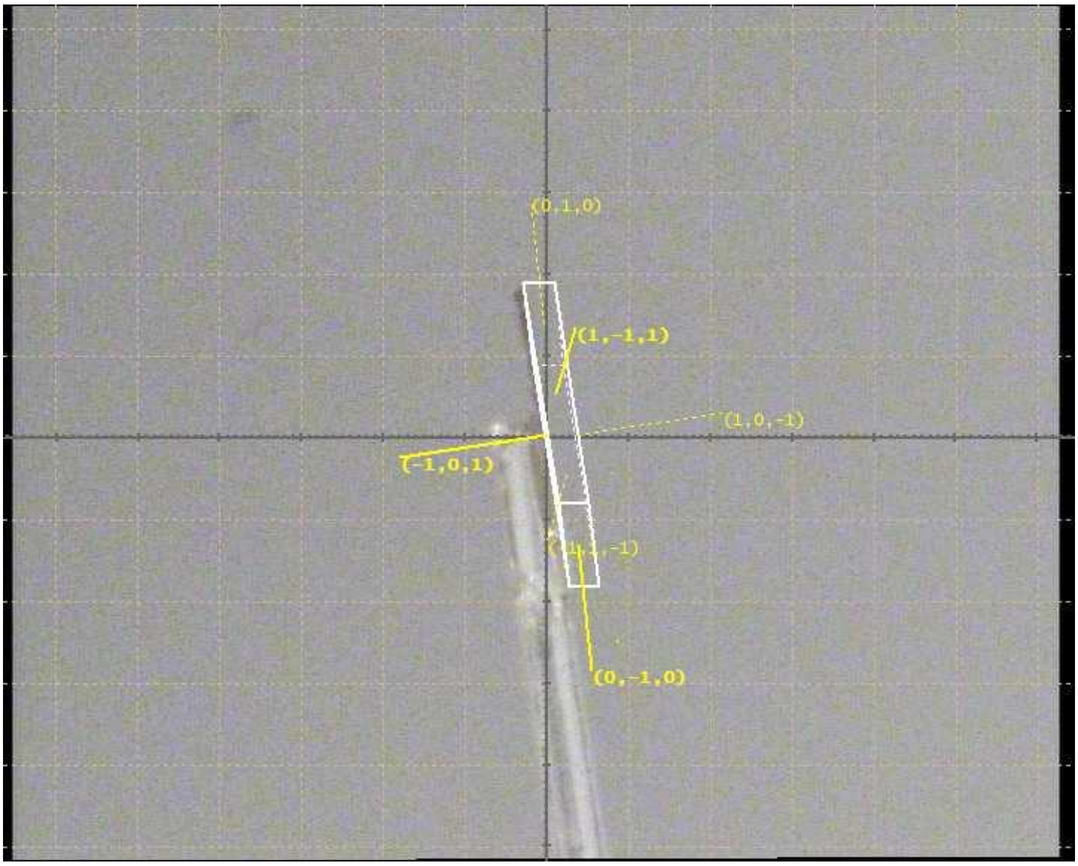

Figure S5 Experimental indexing of Que-Im Cocrystal.

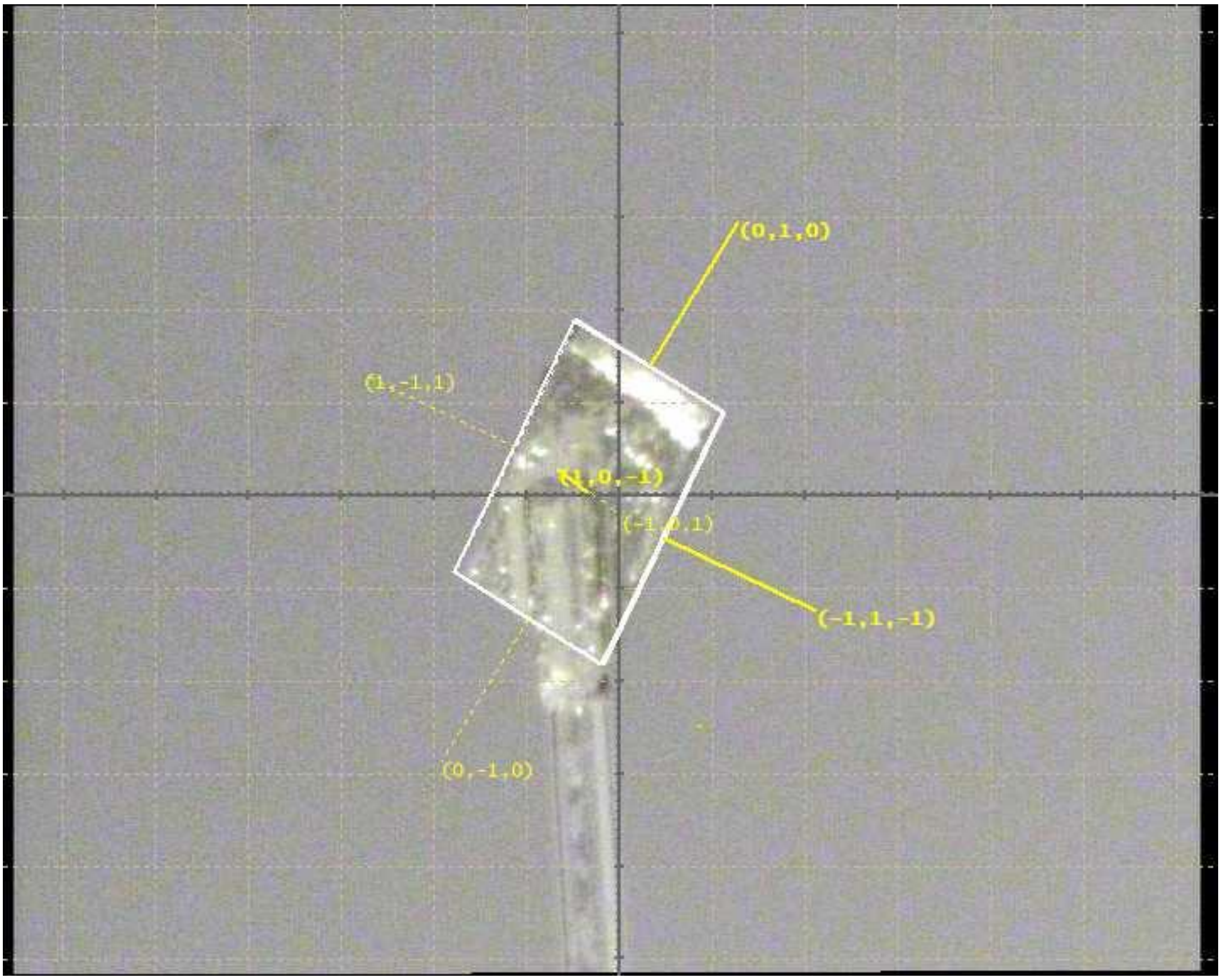

Figure S6 Experimental indexing of Que-Im Cocrystal.

7. Experimental crystal morphology

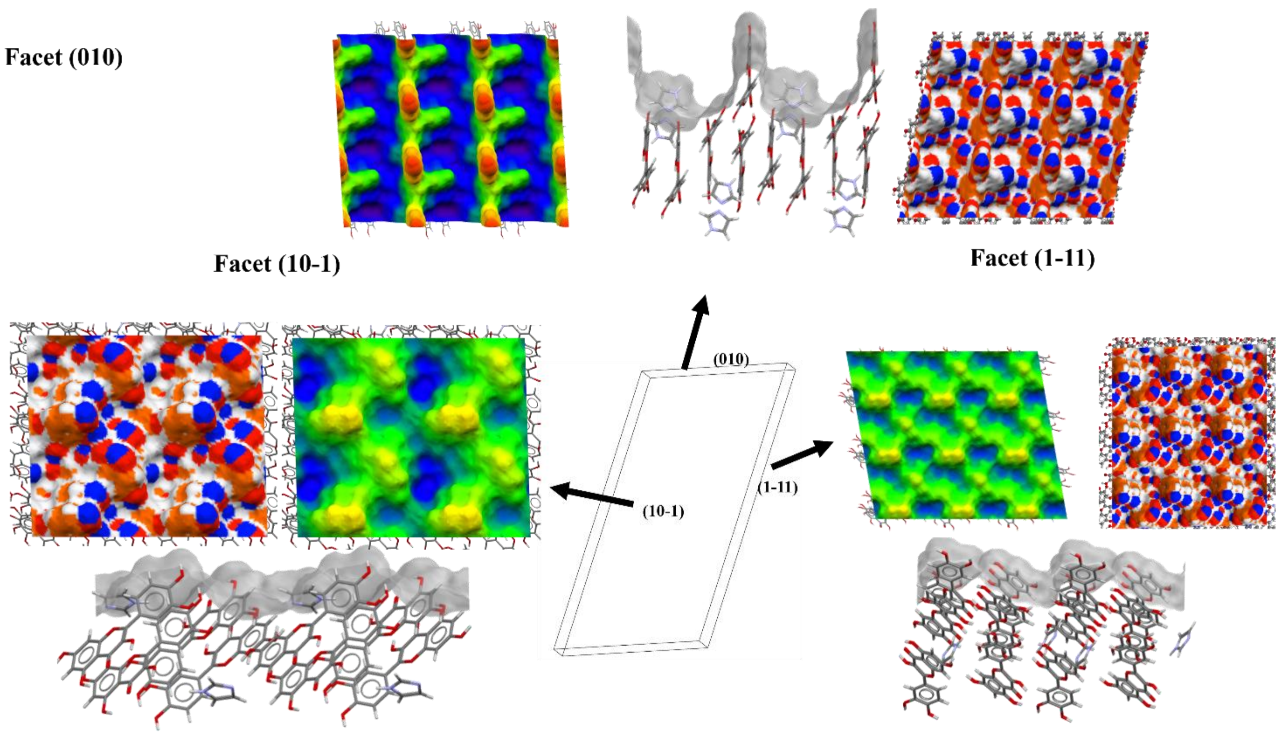

Figure S7. Attachment Energy model of Que-Im experimental morphology. Surface termination is represented for each crystal facet. The terminations of the facets are shown inside view for each facet. The atom properties are represented as H-bond donors (blue). H-bond acceptors (red). aromatic bonds (orange). Surface topology and rugosity comparison for Que-Im: the average plane (green). region above the average plane (yellow and red). and region below the average plane (blue).

Table S8. Crystal habit parameters of Que-Im crystal simulated.

(h k l)	Multiplicity	d_{hkl} (Å)	Total E_{att} (kJ mol^{-1})	Area %
0 0 1	2	11.72	-41.63	32.8
0 1 -1	2	10.05	-53.39	21.0
1 0 0	2	9.73	-88.65	5.0

0 1 0	2	9.79	-71.96	8.5
1 -1 0	2	9.54	-63.37	15.0

8. Geometry optimization

The geometry optimization was performed in Materials Studio 2021 using the Forcite module and the Dreiding (2) forcefield for structural minimization. The Dreiding is a generic force field commonly utilized for organic molecules. It used general force constants based simply on hybridization considerations rather than specific parameters dependent on the interactions of atoms involved in the bonds and angles. The changes in unit cell parameters after geometry optimization are the follow:

Que-Im Optimized : *a* 11.315 (+3.2 %) . *b* 12.620 (+4.0 %). *c* 13.423 (+2-6 %). α 118.816° (+3.5 %. β 88.987° (- 4.6%). γ 120.431° (+6.2 %)

---- Geometry optimization parameters ----

Algorithm : Smart

Convergence tolerance:

Energy : 0.0001 kcal/mol

Force : 0.005 kcal/mol/A

Stress : 0.005 GPa

Displacement : 5e-005 A

Maximum number of iterations : 500

External pressure : 0 GPa

Motion groups rigid : YES

Optimize cell : YES

---- Energy parameters ----

Forcefield : Dreiding

Charges : Charge using Gasteiger

Electrostatic terms:

Summation method : Ewald

Accuracy : 0.0001 kcal/mol

Buffer width : 0.5 A

van der Waals terms:

Summation method : Atom based

Truncation method : Cubic spline

Cutoff distance : 15.5 A

Spline width : 1 A

Long range correction : YES

Buffer width : 0.5 A

Hydrogen bond terms:

Summation method : Atom based

Truncation method : Cubic spline

Cutoff distance : 4.5 A

Spline width : 0.5 A

Buffer width : 0.5 A

9. **Powder X-ray diffraction preferential orientation on powder disk**

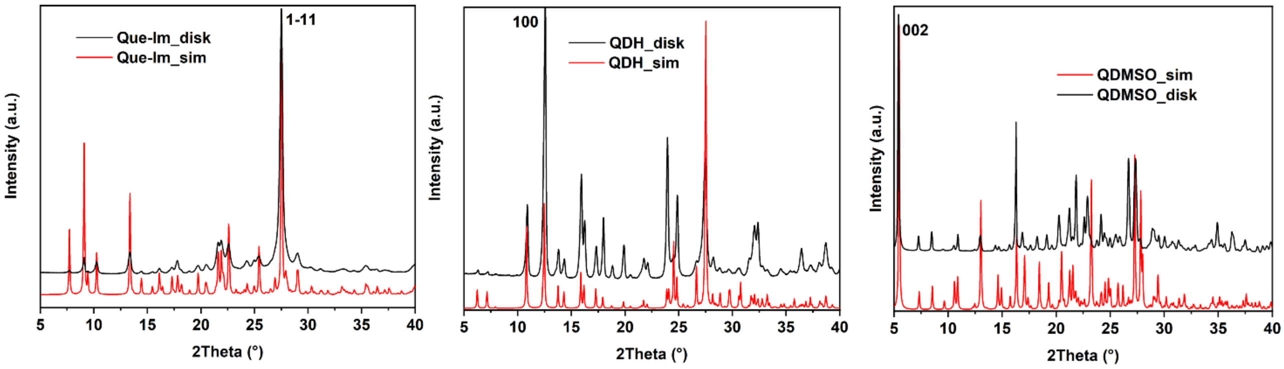

Figure S8. PXRD comparison of Que-Im (left), QDH (center), QDMSO (right) contact angle disks with the simulated pattern.
